# Mesocarnivore sensitivity to natural and anthropogenic disturbance leads to declines in occurrence and concern for species persistence

**DOI:** 10.1002/ece3.70043

**Published:** 2024-07-21

**Authors:** Laken S. Ganoe, Amy E. Mayer, Charles Brown, Brian D. Gerber

**Affiliations:** ^1^ Department of Natural Resources Science University of Rhode Island Kingston Rhode Island USA; ^2^ Fish and Wildlife Division Rhode Island Department of Environmental Management West Kingston Rhode Island USA

**Keywords:** disturbance, mesocarnivore, occupancy dynamics, persistence

## Abstract

Understanding mesocarnivore responses to both natural and anthropogenic disturbance is crucial for understanding species' potential to maintain landscape persistence into the future. We examined the response of five mesocarnivore species (bobcat, coyote, fisher, gray fox, and red fox) to both types of disturbances and climatic conditions. The Northeastern U.S. has experienced multiple large‐scale disturbances, such as a mass defoliation event following larval spongy moth outbreak and high densities of infrastructure that divide the natural landcover into roadless zones where these species inhabit. Using dynamic occupancy models in a Bayesian framework, we aimed to (1) examine variation in species' responses over a 4‐year study by estimating variation in site‐level occupancy, colonization and extirpation of each species in the state of Rhode Island relative to natural disturbance (i.e., defoliation event), anthropogenic disturbance (i.e., parceling of natural landcover bounded by roads, distance to roads), and climate (i.e., seasonal precipitation) and (2) compare current occurrence trends to predicted asymptotic occupancy to identify key variables contributing to distribution instability. Our findings indicated declines in the occurrence of both fox species, and fisher. There was variation in mesocarnivore response to disturbance among the species. We found gray fox and fisher occupancy dynamics to be sensitive to all forms of disturbance and coyote occurrence was positively associated with anthropogenic disturbance. Although bobcat and red fox were predicted to respond positively to future climate scenarios, fisher and gray fox were not, and persistence of fisher and gray fox in a landscape of disturbance relies on large areas with high forest and shrubland cover. With the wide‐spread spongy moth outbreak across much of southern New England, our findings indicate that efforts to conserve forested lands may be crucial in maintaining the persistence of several mesocarnivore species in this region experiencing large‐scale disturbance.

## Graphical abstract

We examined the response of five mesocarnivore species (bobcat, coyote, fisher, gray fox, and red fox) to both types of disturbances and climatic conditions using dynamic occupancy models. There was variation in mesocarnivore plasticity to disturbance among the species. Our findings indicated declines in the occurrence of both fox species, and fisher.

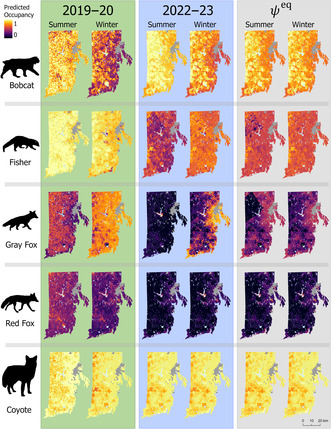

## 1 INTRODUCTION

Wildlife species distributions are affected by both natural and anthropogenic disturbance (Holt & Keitt, 2000; Rio‐Maior et al., 2019; Woodroffe & Ginsberg, 1998). Natural disturbances such as wildfire, climate shifts, and species invasions can alter habitat availability and connectivity, or create phenological mismatch (Boone & McCleery, 2023; Holt & Keitt, 2000; Pozzanghera et al., 2016). Anthropogenic disturbances like urbanization, logging, and agriculture put additional stressors on populations by creating novel environments (Holt & Keitt, 2000; Tuomainen & Candolin, 2011). The plasticity in a species response to disturbance can facilitate species persistence; however, the rapid changes in the landscape and climate in the past century often surpass a species' behavioral plasticity (i.e., ability to adjust behavior in response to a stimuli) or even thermal tolerances potentially resulting in extinction (Pigliucci, 2001; Tuomainen & Candolin, 2011).

Across the globe, anthropogenic disturbance in the form of urban infrastructure and roads are major sources of rapid landscape change (Napton et al., 2010; Plieninger et al., 2016; Raiter et al., 2018). The creation of roads results in changes to landscape configuration by creating zones of natural landcover (Hansen et al., 2005) that are bounded by roads and vary in size and composition (i.e., landcover types), with higher road densities resulting in smaller zones with less natural landcover (Hansen et al., 2005). Smaller zones, however, can be important by contributing to habitat connectivity (Strittholt & Dellasala, 2001). For wide‐ranging carnivores living in areas with high road densities, limited habitat connectivity results in species using many smaller zones of lesser quality habitat to facilitate movement between larger, more suitable zones (Strittholt & Dellasala, 2001). Although roads can be risky for animals (e.g., vehicular strike), roads can also provide beneficial food sources incidentally via roadkill. Road edges and infrastructure provide habitat for abundant small mammal communities that mesocarnivores, like gray fox (*Urocyon cinereoargenteus*), red fox (*Vulpes vulpes*) and coyote (*Canis latrans*) are known to benefit from (Adams & Geis, 1983; Gompper, 2002; Ruiz‐Capillas et al., 2021). Natural disturbance can also provide incidental food sources for mesocarnivores as storm events and natural tree mortality can create habitat for invertebrate and small mammal communities that support mesocarnivore diets (Carey & Johnson, 1995; Kirkland, 1990). Understanding the unique responses of mesocarnivores to various types of disturbance aids in assessing species' persistence in a rapidly changing environment.

Studying carnivore response to disturbance under climate projections is important in anticipating future changes to proactively direct conservation and management actions (Gerber & Kendall, 2018; Williams et al., 2009). Increasing global temperatures, changes in intensity of storm events, and drastic changes in precipitation are expected in the next century regardless of emissions scenarios (Collins et al., 2013; Tang & Beckage, 2010). In the northeastern United States, climate scenarios predict changes in precipitation and temperature will result in deciduous forest replacement of existing coniferous forests (Janowiak et al., 2018); these changes are expected to result in species distribution shifts (Chamberlain et al., 2013). Although some species, such as small mammals, may respond positively to increased precipitation in the form of rain, carnivores like marten (*Martes americana*) and lynx (*Lynx canadensis*) are sensitive to changes in winter precipitation (i.e., snow) and populations are expected to contract in response to decreased snowfall (Carroll, 2007; Meserve et al., 2011; Pozzanghera et al., 2016). Changes in regional climate in the form of precipitation and storm severity compound with other natural (i.e., invasions, wildfire) and anthropogenic (i.e., urbanization, roads) disturbances creating an uncertain future for many species.

In the northeast United States, apex predators have been extirpated from the landscape leaving space for the mesocarnivore community (e.g., bobcat (*Lynx rufus*), coyote, fisher (*Pekania pennanti*), red fox, and gray fox) to expand (Prugh et al., 2009). Mesocarnivore populations can reach higher densities in the absence of apex predators (i.e., mesocarnivore release), particularly in disturbed areas where smaller predators are more efficient at exploiting prey than their larger counterparts (Crooks & Soule, 1999; Prugh et al., 2009; Vance‐Chalcraft et al., 2007). Several mesocarnivore species, like coyotes and fisher, have seen rapid increases in range distributions in the northeastern U.S. in response to both a lack of apex predators and anthropogenic disturbance (Gompper, 2002; Kontos & Bologna, 2008; Lapoint et al., 2015; Lewis et al., 2012; Moncrief & Fies, 2015). In areas with no apex predators, species dietary niches broaden in response to areas of higher human‐use (Schuette et al., 2013; Smith et al., 2018). Across their range, there is much overlap in mesocarnivore niches and variation in sensitivity to disturbance, with some species like bobcat and gray fox being more sensitive to habitat loss and road disturbance than others, like coyote (Carroll et al., 2019; Lovallo & Anderson, 1996; Smith et al., 2018).

To better understand the persistence of mesocarnivores on the landscape, we investigated the effects of natural and anthropogenic disturbance, and climate on mesocarnivores in Rhode Island, USA. Rhode Island has experienced multiple large‐scale disturbances. As the second most densely populated state in the United States, Rhode Island has experienced abundant anthropogenic disturbances, including high road densities and forest loss (Jeon et al., 2014; US Census Bureau, 2012). Additionally, between 2015 and 2017 southern New England experienced a mass defoliation event due to a spongy moth (*Lymantria dispar*) larval outbreak that affected almost 4400 km^2^ of forest (Pasquarella et al., 2018). Following the event, increased oak tree (*Quercus* sp.) mortality led to drastically altered leaf litter, mast production, and light availability in affected areas (Pasquarella et al., 2018).

We anticipated varying responses to each type of disturbance across the Rhode Island mesocarnivore guild (Appendix S1). Our objectives were to (1) examine species' variation in estimated responses of site‐level occupancy, colonization and extirpation of five mesocarnivore species (i.e., bobcat, coyote, fisher, gray fox, and red fox) in the state of Rhode Island to natural disturbance (i.e., defoliation event), anthropogenic disturbance (i.e., parceling of natural landscape bounded by roads, distance to roads), and climate (i.e., seasonal precipitation), and (2) compare current occurrence trends to predicted asymptotic occupancy to identify key variables contributing to distribution instability. We hypothesized that forest dynamics related to moth damage and succession would influence fisher the most as they prefer high canopy cover (Kordosky, Gese, Thompson, Terletzky, Neuman‐Lee, et al., 2021; Kordosky, Gese, Thompson, Terletzky, Purcell, & Schneiderman, 2021; Sauder & Rachlow, 2015). We anticipated that the effects of anthropogenic disturbance in the form of roads would have the most impact on the fox species with gray fox avoiding roads and red fox benefiting from roads (Adams & Geis, 1983; Carroll et al., 2019; Riley, 2006; Ruiz‐Capillas et al., 2021). Additionally, we expected seasonal variation in site occupancy related to differences in seasonal home range size and movement for all species (Cypher, 2003; Hersteinsson & Macdonald, 1982; Mayer et al., 2021; Powell, 1993) except coyote, since home ranges of coyotes living near urbanization remains constant across seasons (Crête et al., 2001; Gehrt et al., 2009). Lastly, we hypothesized that coyote would have high landscape occurrence and this would remain stable across the study period due to their ability to acclimate to changing conditions and their generalist nature.

## 2 METHODS

### 2.1 Study area

This study was conducted in Rhode Island, USA, the second most densely populated state with a human density of 410 people/km^2^ (US Census Bureau, 2012). The landcover across this study area is primarily composed of forest (46.6%), development (30.8%), and woody wetlands (11.6%). Rhode Island borders the Atlantic Ocean to the south and east, which is where the most intensive human development occurs. Road density across the study area ranged from 0 to 28.3 km of total road length per km^2^.

### 2.2 Data collection

**FIGURE 1:**
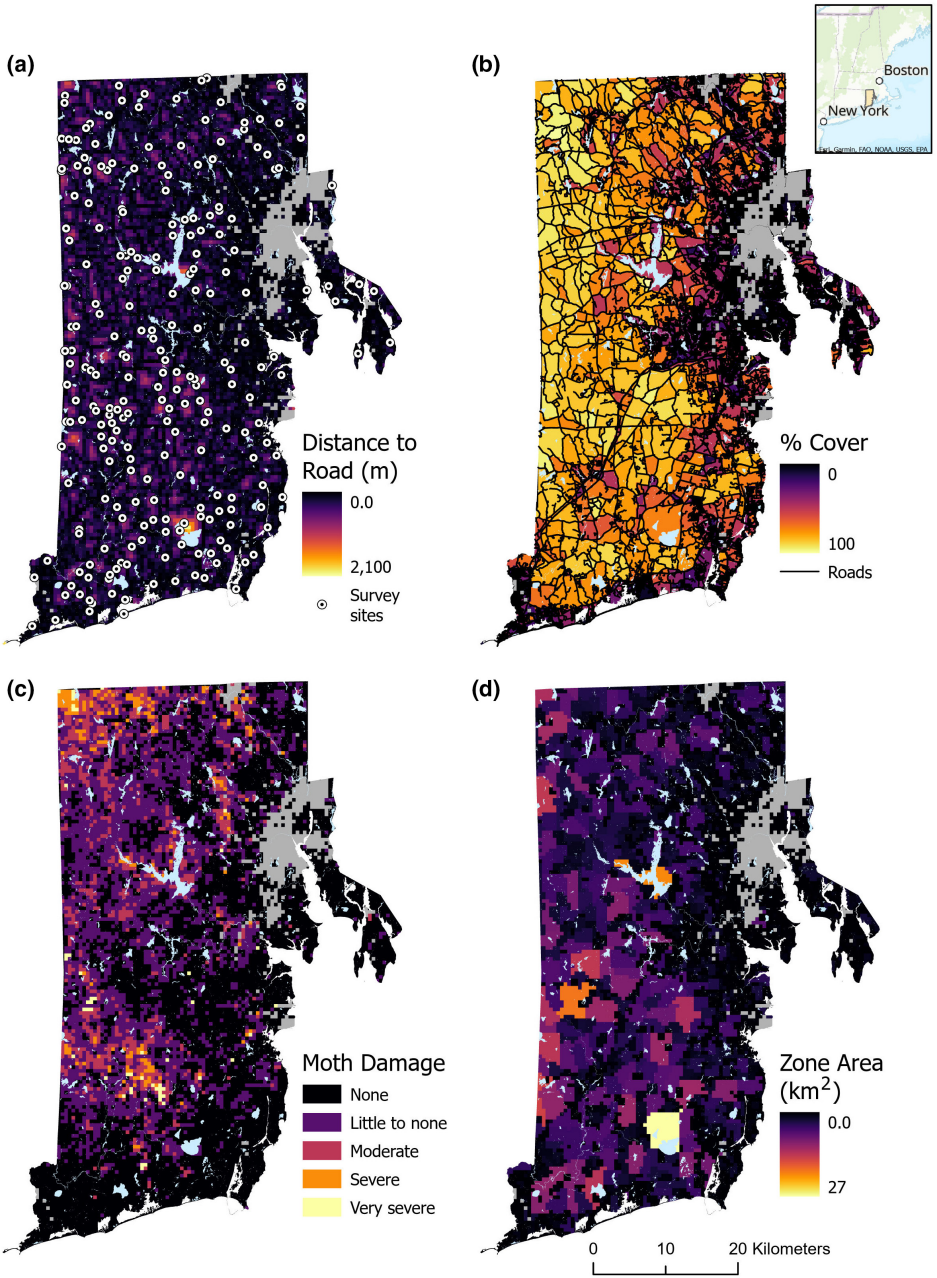
Maps of variables and survey locations across the study site in Rhode Island, USA.

We sampled mesocarnivores using trail‐cameras deployed across the state of Rhode Island, USA (41.5801° N, 71.4774° W) for four winters (November–March) and four summers (June–October) from 2019 to 2023 (Figure 1a; Table 1). The state was gridded into 1 km^2^ square cells to reduce spatial autocorrelation among sites while allowing for capture of fine‐scale landscape variation. Sites were selected to be sampled from the grid through stratified random sampling to ensure representation of major landcover features (i.e., forest, development, road density). At each site, we deployed two motion‐triggered trail cameras (Bushnell Trophy Cam, Bushnell Outdoor Products, Overland Park, KS, USA, or Browning Strike Force Pro XD, Browning, Morgan, UT, USA). Cameras were placed near a random point where access permitted and camera placement maximized detection (e.g., rock walls, game trails, fallen logs). Within a site, the two cameras were between 50 and 100 m apart. A commercial lure (“Caven's Gusto”; Minnesota Trapline Products, Pennock, MN, USA) was applied to a nearby tree at each camera during deployment to increase detection of predators in the area.

**TABLE 1:** Summary of camera trapping surveys by season and year in Rhode Island, USA with the total number of sites deployed with paired cameras and date range.

Year	Winter		Summer	
	# sites	Dates	# sites	Dates
2019	–	–	100	June 10—Sep 30
2020	100	Dec 2—Mar 14	100	June 8—Sep 18
2021	200	Nov 14—Mar 10	240	May 26—Sep 10
2022	240	Nov 1—Feb 14	240	May 29—Sep 5
2023	100	Nov 28—Mar 4	–	–

We started sampling in 2019 with 100 sites carried over from a long‐term bobcat study (Mayer et al., 2022). Sample size was increased by 100 additional sites in winter of 2021 and then again in the following summer by an additional 40 sites (Appendix S1: Figure S1). In winter of 2023, sites were reduced back to the original 100 survey locations (Table 1). We sampled within each season for a minimum of 12‐weeks (Appendix S1). Photo data was organized and processed in the photo database Camelot (Hendry & Mann, 2018). Detections were considered independent if at least 20 min had elapsed between photos of each species at each site (Burton et al., 2015; Mayer et al., 2022).

### 2.3 Data analysis

#### 2.3.1 Covariate selection

We defined seven variables across the three categories of interest for this analysis: natural disturbance, anthropogenic disturbance, and climate (Table 2). A recent landscape‐scale natural disturbance in the study area was the mass defoliation event caused by the spongy moth (*Lymantria dispar*). We incorporated forest effects from this event using measures of severity of damage and time since initial disturbance in 2015 as variables (Figure 1c). Spongy moth damage data were obtained from 2017 Landsat imagery that defined four severity categories of changes in Greenness values from “slight change” to “very large change” (Pasquarella et al., 2018).

**TABLE 2:** Variables used in dynamic occupancy modeling of mesocarnivores in Rhode Island, USA with associated category of interest they represent.

Variable	Description	Category	*ψ*_1_	*γ*	*ϵ*	*p*
Moth	Median moth damage within 200 m of survey site	Natural	✓	✓	✓	
TSD	Time since defoliation event in years	Natural		✓	✓	
Cover	Percent cover^a^ within zone between roads	Anthro	✓	✓	✓	✓
Road_Dist	Distance from survey site to nearest road	Anthro	✓			
Zone_Size	Area (km^2^) of zone between roads	Anthro		✓	✓	✓
Season	Climatic season—winter/summer	Climate		✓	✓	
Precip	Seasonal precipitation (summer = rain, winter = snow)	Climate		✓	✓	

*Note*: Check marks indicate whether the variable was included on a specific parameter—initial occupancy (*ψ*_1_), colonization (*γ*), extirpation (*ϵ*), detection (*p*).

^a^ Cover is defined as National Landcover Database categories that include forests, shrubland, and woody wetlands.

**FIGURE 2:**
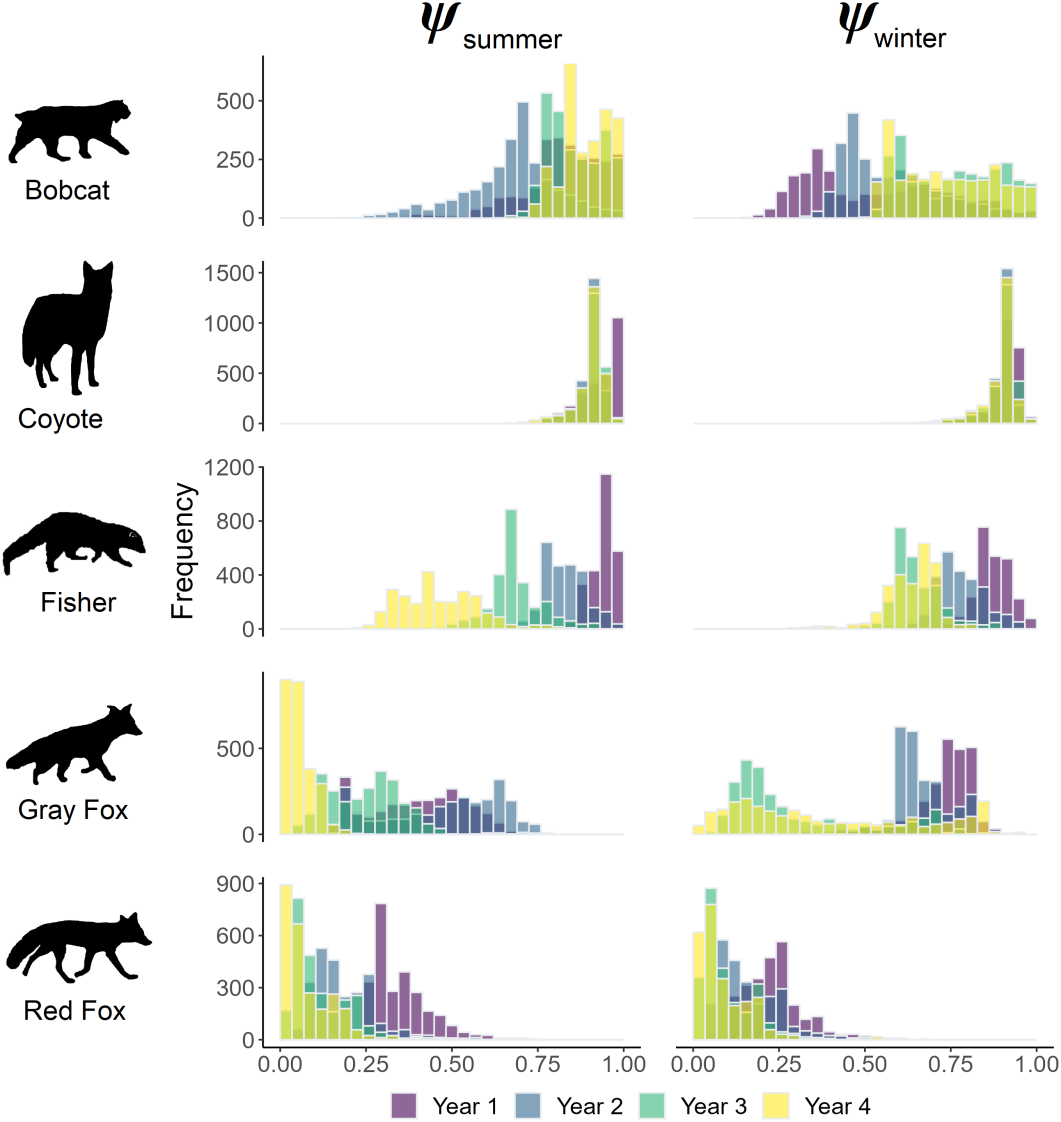
Histograms of median posterior site occupancy probabilities for mesocarnivore species across the prediction grid by season and year. Mean change in occupancy probabilities for each species and season were as follows: Bobcat (λ_summer_ = 1.05, λ_winter_ = 1.44), coyote (λ_summer_ = 0.96, λ_winter_ = 0.99), fisher (λ_summer_ = 0.50, λ_winter_ = 0.74), gray fox (λ_summer_ = 0.12, λ_winter_ = 0.43), and red fox (λ_summer_ = 0.14, λ_winter_ = 0.29).

The landscape in Rhode Island has multiple large‐scale anthropogenic disturbances but is particularly impacted by infrastructure in the form of roads. Roads fragment the landscape into a mosaic of parcels, of which we defined the area between roads as zones (Figure 1b). The size and composition of zones may limit or promote mesocarnivore occurrence for species whose life history traits require space (e.g., home range size) and cover (e.g., denning). Using road layers from RIGIS (RIGIS, 2016) and landcover classes (Dewitz & Survey, 2021), we classified anthropogenic disturbance in three ways; (1) distance from a site to the nearest road that is two‐lane or larger (Road_Dist; Figure 1a), (2) percent vegetation cover (defined as 2019 National Landcover Database [NLCD] categories that include forests, shrubland, and woody wetlands) in each zone (Figure 1b) where lower amounts of cover are associated with higher human disturbance in the form of development and agriculture, and (3) total area of a zone (Figure 1d; Table 2).

Since seasonal precipitation affects prey assemblages and thus their predators (i.e., mesocarnivores), we included seasonal precipitation to investigate responses to different intensities of rain and snow (Meserve et al., 2011; Pozzanghera et al., 2016). Effects of seasonal precipitation are interpreted as follows: responses in spring transition periods (between sampling seasons winter to summer) relate to the preceding winter snowfall while responses in autumn transition periods (between sampling seasons summer to winter) relate to the preceding summer rainfall. Additionally, we wanted to investigate how current mesocarnivore responses might affect persistence as climate predictions anticipate increased precipitation in this region. Precipitation data in the form of daily rain and snowfall were obtained through the Applied Climate Information System of the National Weather Service and totaled for the duration of each sampling season as defined above (NOAA, 2023).

#### 2.3.2 Hierarchical dynamic occupancy modeling

We fit models to each species' data separately in a Bayesian framework using dynamic occupancy models (MacKenzie et al., 2003) with diffuse priors available in the R package “ubms” (Kellner et al., 2022) in R version 4.1.1 (R Core Team, 2023). We considered the same general model structure for each species because we were interested in understanding the combined effects of the main landscape‐scale drivers (natural and anthropogenic disturbance) and the consistency of these relationships across the mesocarnivore community. All continuous variables were mean centered and scaled to allow direct comparison of coefficients as one unit change in standard deviation of a covariate value. For each species, we estimated site‐level initial occurrence (*ψ*_1_), colonization (*γ*), extirpation (*ϵ*), and detection (*p*) (MacKenzie et al., 2003). We modeled *ψ*_1_ and *p* using additive combinations of moth damage, cover, with distance to road, and cover with zone size, respectively (Table 2). To accommodate for unmodeled site‐level heterogeneity in detection, we also included a site‐level random effect in each model. For colonization probability (γ), we considered variables to vary by site (*i*) and season/year (*t*) using additive and pair‐wise interaction combinations as,

(1) $$\begin{array}{c} \text{logit}\left(\gamma_{i,t}\right)=\beta_{0}+\beta_{1}{\text{precip}}_{i,t}+\beta_{2}{\text{season}}_{t}+\beta_{3}{\text{precip}}_{i,t}*{\text{season}}_{t}\\ +\beta_{4}\text{zone}\;{\text{size}}_{i}+\beta_{5}{\text{cover}}_{i}+\beta_{6}\text{zone}\;{\text{size}}_{i}*{\text{cover}}_{i}+\beta_{7}{\text{moth}}_{i}+\beta_{8}{\mathrm{TSD}}_{t}\\ +\beta_{9}{\text{moth}}_{i}*{\mathrm{TSD}}_{t}. \end{array}$$

Extirpation probability followed the same form as that in Equation 1 with a separate set of coefficients defined on $\text{logit}\left(\epsilon_{i,t}\right)$. From the dynamic parameters, we derived site occupancy for each subsequent primary sampling period (*ψ*_*t*_) as well as site turnover probability ($\overset{´}{\tau_{t}}$) as the probability of a site changing occupancy status from one season to the next (MacKenzie et al., 2017, p. 362). Higher turnover probabilities indicated lower site fidelity and higher variation in site occupancy between seasons.

For each model, we fit three parallel chains using random starting values and a burn‐in‐period of 2500 iterations, followed by 5000 Markov Chain Monte Carlo samples. We assessed parameter convergence visually by inspecting trace‐plots and using the *R*‐hat statistic where we found all parameters showed convergence with *R‐*hat values near 1 (Gelman et al., 2004). We made inference based on estimated coefficient size (reported as the posterior median, *β*, where a large effect is considered >1 or <−1) and the probability that a coefficient was different than zero (p_pos; derived as the number of posterior samples >0). Strong support was defined as ≥0.90 or ≤0.10 probability of coefficients being greater than zero (positive and negative support, respectively), and moderate support was defined as ≥0.70 and ≤0.90 or ≤0.3 and ≥0.10 probability of coefficients being greater than zero (positive and negative support, respectively). The two seasonal transition periods modeling γ and *ϵ* were summer‐to‐winter (autumn) and winter‐to‐summer (spring), which were defined using dummy coding as factor levels 0 and 1, respectively. Positive seasonal effects are interpreted as higher response values (e.g., γ probability) occurring in spring than in autumn.

#### 2.3.3 Prediction

We predicted species occurrence throughout Rhode Island by season using a 1 km^2^ grid overlaid across the study area. Within each grid cell, variables were extracted as defined above for the survey sites with the exception of moth damage, which was calculated as the median moth damage within each grid cell. Since our sampling design did not encompass areas with intense development (i.e., major cities), we removed cells in the prediction grid where over 40% of the cell was defined as belonging to the high development landcover class from the NLCD which was the upper limit covered by our survey sites. We also removed water bodies from the prediction grid to accurately represent terrestrial species occurrence. We calculated the rate of change in seasonal occupancy across the prediction grid from the first to last year of the study as $\left(l_{t}=\frac{\psi_{t}+1}{\psi_{t}}\right)$ (MacKenzie et al., 2017). Additionally, we assessed trends in seasonal occupancy by calculating, *c*_s_ (summer) and *c*_w_ (winter), as the proportion of grid cells that experienced a decline (i.e., $\;l_{t}<1$) over the course of the study for each season (i.e., summer, winter). Lastly, we assessed occupancy stability by predicting the stable state occurrence defined as the equilibrium of γ and *ϵ* in each grid cell *i* where $\left(\psi_{i}^{\mathrm{eq}}=\frac{\gamma_{i}}{\gamma_{i}+\epsilon_{i}}\right)$ (MacKenzie et al., 2017). To capture seasonal dynamics, we predicted two separate stable state occurrences, one for summer and one for winter. The stable state allowed us to identify the expected distribution when occurrence dynamics are not fluctuating. Comparing the stable state to our observed occurrence patterns allowed us to make inferences on the trends of each species distributions.

## 3 RESULTS

### 3.1 Camera trapping

Cameras were deployed for 47,677 total trap nights over the duration of the study capturing over two million photos. Of the mesocarnivore species, coyote were detected the most often (*n* = 3956 detections), followed by fisher (*n* = 2526 detections), and red fox (*n* = 1288 detections). Gray fox and bobcat had the least number of detections at camera sites (*n* = 831, *n* = 520 detections, respectively). Across species, coyotes had the highest naïve occupancy (range = 0.7–0.88), whereas gray foxes had the lowest (range = 0.05–0.31) in each season (Table 3).

**TABLE 3:** Naïve occupancy for each species, by season (S = summer, W = winter) and year of survey.

	Bobcat		Coyote		Fisher		Gray Fox		Red Fox	
	S	W	S	W	S	W	S	W	S	W
2019–20	0.21	0.20	0.70	0.88	0.65	0.79	0.12	0.31	0.14	0.43
2020–21	0.17	0.23	0.78	0.73	0.67	0.64	0.14	0.17	0.20	0.31
2021–22	0.19	0.21	0.72	0.72	0.50	0.63	0.10	0.15	0.16	0.38
2022–23	0.29	0.31	0.76	0.86	0.38	0.47	0.05	0.13	0.16	0.38

### 3.2 Occupancy models

**FIGURE 3:**
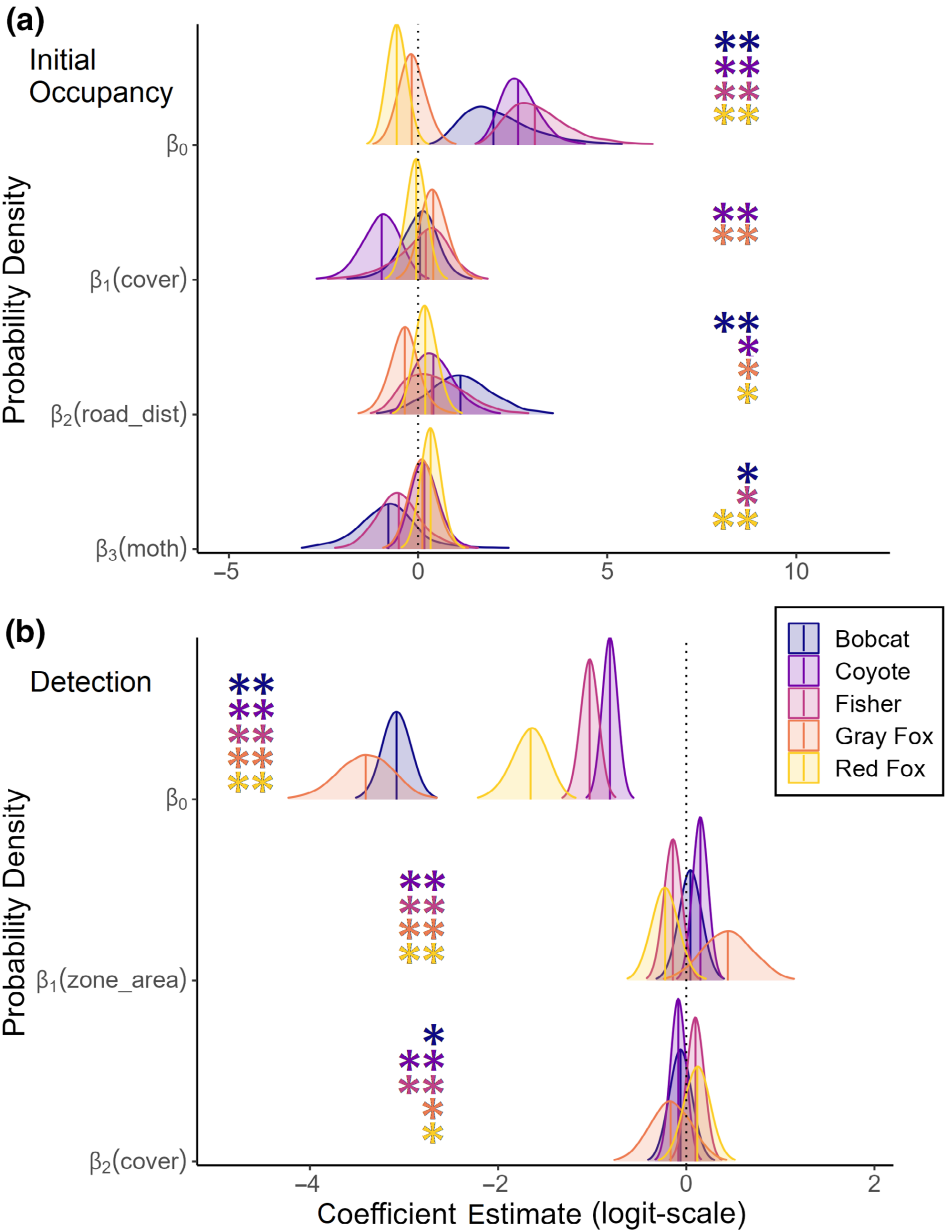
Occupancy (a) and detection (b) probability posterior distributions of coefficient estimates by species. Strong support (**) was defined as ≥0.9 or ≤0.1 probability of coefficients being greater than zero (positive and negative support, respectively), and moderate support (*) was defined as ≥0.7 or ≤0.3 probability of coefficients being greater than zero (positive and negative support, respectively).

For all species except for coyote and bobcat, site‐level occupancy estimates declined in at least one season from the beginning to end of the study (Figure 2). Our models indicated that at least one variable was moderately or strongly supported as impacting *ψ*_1_ for all species (Figure 3a; see Appendix S2: Table S1 for all coefficient estimates). Detection probability was associated with zone area for all species except for bobcat, and our models indicated site‐level random effects accounted for much of the variation in detection (Figure 3b; Appendix S2). Colonization and extirpation probabilities were largely associated with seasonal effects. Site turnover varied by species and in response to moth damage, time since disturbance, zone area, and cover. As there was variation across species' associations with disturbance, we will further highlight individual species model results with strong or moderate support related to our predictions (Table 4).

**TABLE 4:** Descriptions of hypotheses, predictions, and findings by species and disturbance type.

Species	Disturbance	Hypothesis	Predicted response	Findings	Relevant citations
Bobcat	Human	Bobcat are known to be sensitive to anthropogenic disturbances, avoiding roads and human development ‐ thus occurring farther away from roads in more contiguous forested areas. They also need cover to hunt and protect themselves from harsh weather. However, they will use areas with early successional forest that may occur near agricultural areas that may be represented by the larger zones with lower cover. Generally elusive with low detection rates	Avoid roads: Higher colonization in medium to large zones with low cover. Higher extirpation in smaller zones with low cover. Low turnover in large, covered areas	Highest colonization in small zones with low cover. Higher extirpation rates in small zones, regardless of cover. High turnover in small zones or areas with low cover	Bailey (1974), Lovallo and Anderson (1996), Fuller and DeStefano (2003), Riley (2006), Carroll et al. (2019), Mayer et al. (2021, 2022)
	Natural	Bobcat are sensitive to habitat loss, but they do prefer early successional forest and benefit from natural forest disturbances. As moth damaged areas undergo forest succession, we would anticipate bobcat to respond positively to new early successional habitat that is created	More likely to colonize severely moth damaged areas over time	In 2–3 years after disturbance, bobcat were more likely to colonize sites with severe moth damage, but after year 3.5 they became less likely to colonize those areas	Fuller and DeStefano (2003)
	Climate	There is seasonal variation in bobcat home ranges with larger home ranges in winter. They have been known to shrink their movements when there is deep snow, however movements shift to trails which are abundant in our study area	Higher occupancy in winter, but colonization probability will decrease with increased winter snow	Higher occupancy in summer, colonization probability increased with increasing winter snow	Marston (1942), McCord (1974), Litvaitis et al. (1986), Anderson (1987), Lovallo and Anderson (1996), Mayer et al. (2021)
Coyote	Human	Coyote are a highly adaptable species and range distributions have increased in response to anthropogenic disturbance. They also benefit from habitat created near roads for small mammal communities. Pack animals, able to establish larger packs in areas with more space and food	Overall, coyote will respond positively to human disturbance. Little variation in occurrence across state. Higher detection in large zones related to larger pack sizes	Lower turnover in low cover zones, but overall little variation and high occurrence across the state regardless of disturbance	Adams and Geis (1983), Gompper (2002), Ruiz‐Capillas et al. (2021)
	Natural	Highly adaptable species, no evidence that they would have a strong response to defoliation	Lack a strong response to natural disturbance	No response to moth damaged areas	Adams and Geis (1983), Gompper
	Climate	Coyote are known to respond positively to increased small mammal abundance in years with more summer precipitation. In urban areas, coyote home range sizes remain constant across season	Positive response to summer rain, but occupancy will not vary by season	No response to either form of precipitation and no seasonal variation	Gehrt et al. (2009), Meserve et al. (2011)
Fisher	Human	Fisher have expanded their range into areas of anthropogenic disturbance but still rely on cover for hunting and protection. They are also tolerant of some anthropogenic disturbances	Areas of high cover and large zones will have low turnover (will remain occupied) and turnover and detection will be higher in areas with less cover	Lowest turnover in large, high cover zones, and highest turnover in large zones with low cover. Higher detection in areas with more cover	Kelly (1978), Zielinski et al. (2006), Kontos and Bologna (2008), Lofroth et al. (2010), Lewis et al. (2012), Naney et al. (2012), Raley et al. (2012), LaPoint et al. (2013), Moncrief and Fies (2015)
	Natural	Fisher rely on canopy cover for hunting opportunities, and the early successional forests created by moth damage is not ideal habitat for them. Additionally, in areas with tree mortality, fisher stress levels were higher, suggesting less than ideal conditions	Less likely to colonize moth damaged areas over time	High colonization probability in moth damaged areas over time, and higher turnover in severely damaged areas	Kelly (1978), Zielinski et al. (2006), Lofroth et al. (2010), Kordosky, Gese, Thompson, Terletzky, Neuman‐Lee, et al. (2021), Kordosky, Gese, Thompson, Terletzky, Purcell, and Schneiderman (2021)
	Climate	Fisher have seasonal variation in home range sizes and activities. They also avoid areas with deep snow	Occupancy will differ between seasons and fisher will respond negatively to winter snow	Seasonal variation in occupancy and overall negative response to increasing winter snow	Powell (1993), Powell and Zielinski (2003)
Gray Fox	Human	Gray fox are sensitive to habitat loss and road disturbance. They are considered a more forest‐dependent species. When they do use areas with high human densities, their core areas remain entirely within natural areas. They will use mixed agricultural areas, but require adjacent tree cover	Lower turnover in areas with high cover, and higher turnover rates in areas with low cover. Higher detection in larger zones with more cover	Highest turnover rates in small zones with little cover, turnover decreases with increasing cover and zone size. Higher detection in larger zones	Wood (1958), Follman (1973), Hall (1981), Riley (2006), Kowalski et al. (2015), Harrison (1997)
	Natural	Core territories require forested, undamaged areas	Lower occupancy and colonization in areas with moth damage. Higher extirpation rates as time passes in moth damaged areas	In severely damaged areas, gray fox became less likely to extirpate sites over time, however they became more likely to extirpate sites with no to moderate damage	Riley (2006), Kowalski et al. (2015)
	Climate	Gray fox would be likely to respond positively to summer rain since small mammal abundance responds positively. Gray fox have different seasonal home ranges, however this is mediated by prey availability	Occupancy will differ slightly between season, and gray fox will respond positively to summer rain	Seasonal variation in occupancy, and gray fox had higher colonization than extirpation when less than 30 inches of rain	Cypher (2003), Meserve et al. (2011)
Red Fox	Human	Red fox benefit from habitat created near roads for small mammal communities. They also hunt in more edge‐habitat and agricultural areas which makes them easier to detect in those areas	Lower turnover in areas with low to medium cover regardless of zone size and will have higher occupancy close to roads. Higher detection in smaller zones with less cover	Lower turnover in high cover and small low cover zones. Higher occupancy in large zones of low cover. More likely to occupy sites far from roads. Higher detection in smaller zones with less cover	Ables (1975) Adams and Geis (1983), Catling and Burt (1995), Ruiz‐Capillas et al. (2021)
	Natural	Red fox prefer early successional forest and benefit from the creation of those habitats by natural disturbance	Positive response to moth damage with higher colonization in moth damaged areas as time passes	Slight decline in turnover in areas with no moth damage, and increasing turnover in severely damaged areas over time. Higher colonization and extirpation in moth damaged areas over time at different rates	Fuller and DeStefano (2003)
	Climate	Red fox would be likely to respond positively to summer rain since small mammal abundance responds positively. Red fox have different seasonal home ranges, however this is mediated by prey availability and more likely to stabilize in urban environments	No seasonal variation in occupancy, but they will respond positively to summer rain	Annual variation in occupancy, and red fox became more likely to colonize and more likely to extirpate areas with more rain	Hersteinsson and Macdonald (1982), Cypher (2003), Meserve et al. (2011)

#### 3.2.1 Bobcat

**FIGURE 4:**
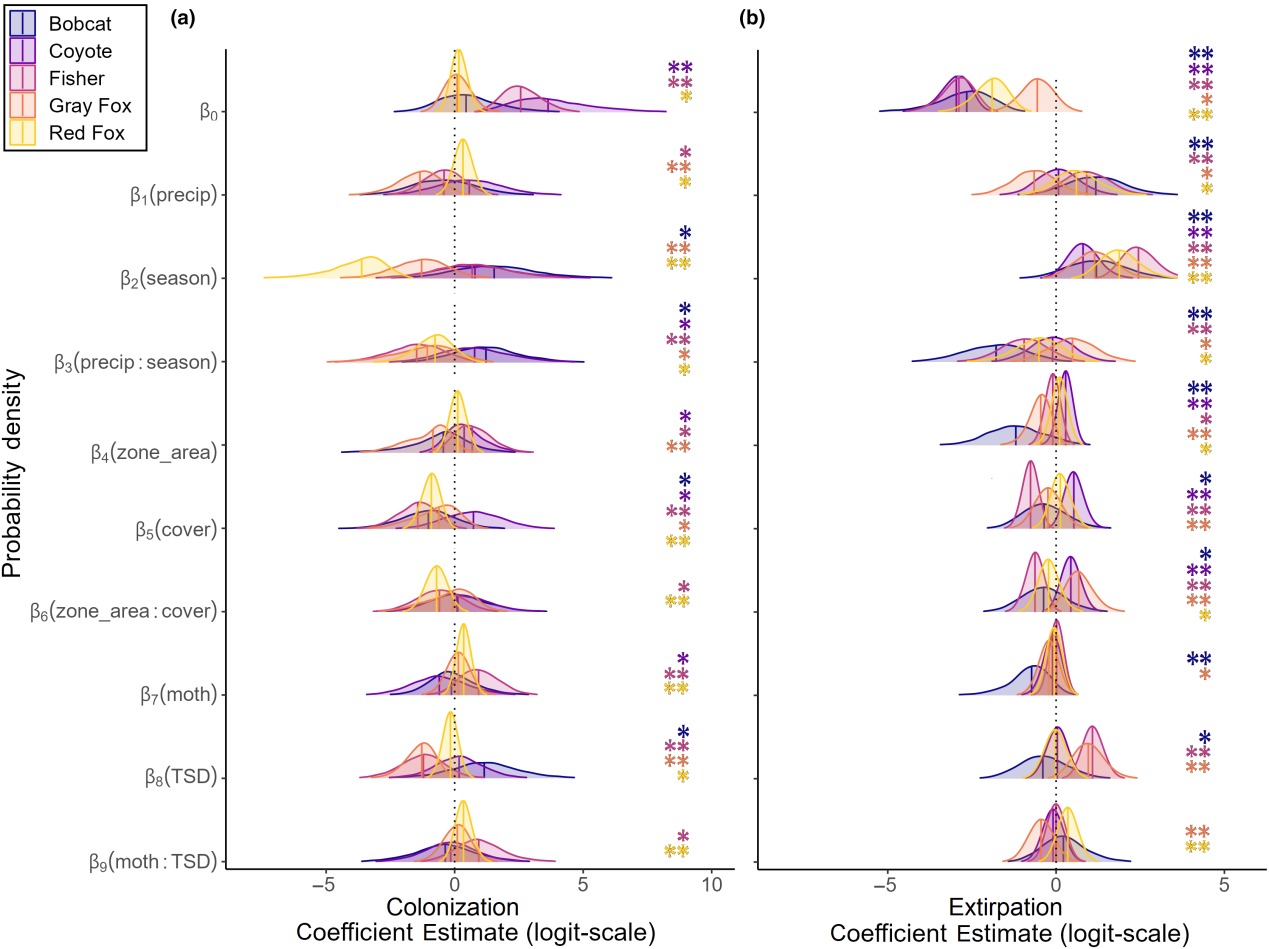
Colonization (a) and extirpation (b) probability (γ,ϵ, respectively) posterior distributions of coefficient estimates by species. Strong support (**) was defined as ≥0.9 or ≤0.1 probability of coefficients being greater than zero (positive and negative support, respectively), and moderate support (*) was defined as ≥0.7 or ≤0.3 probability of coefficients being greater than zero (positive and negative support, respectively).

**FIGURE 5:**
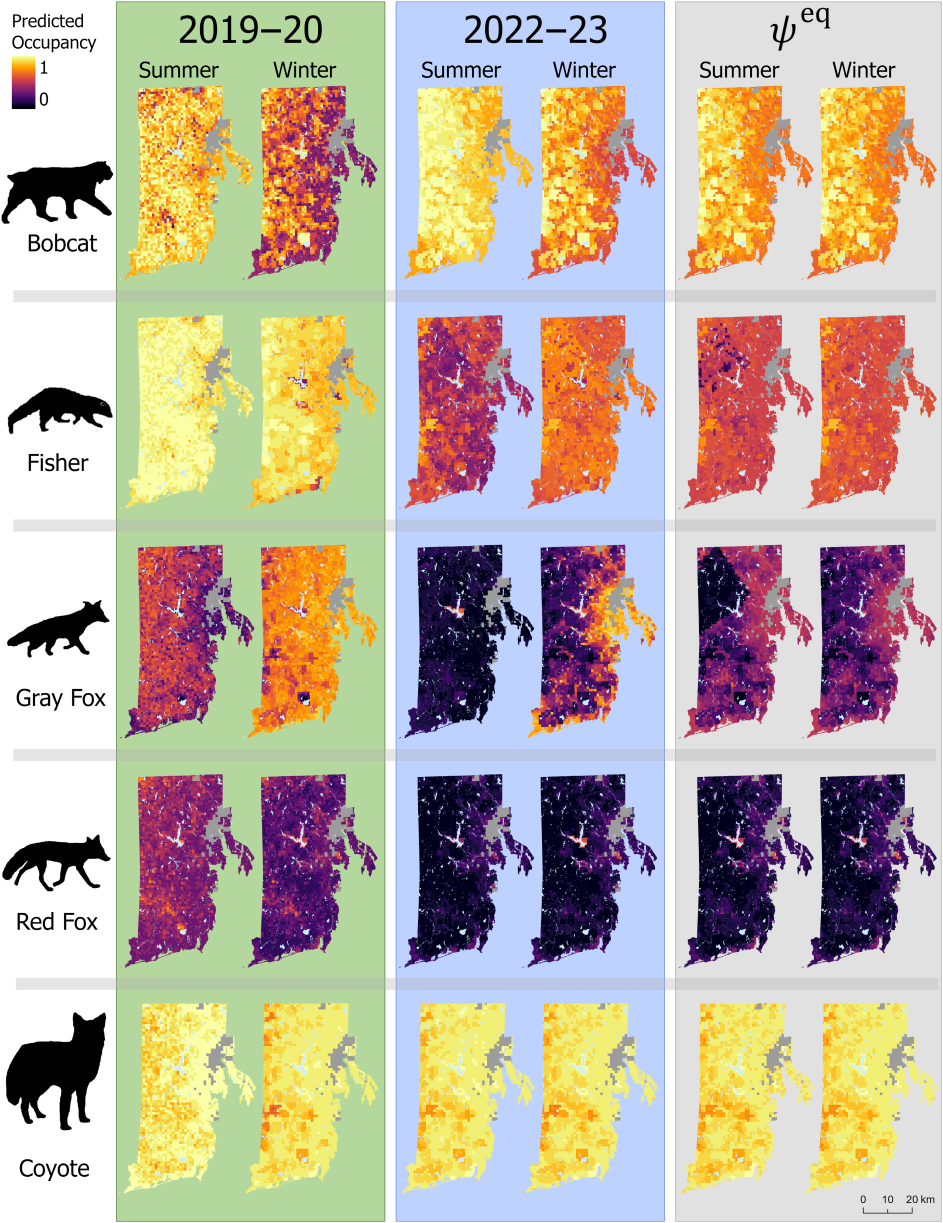
Maps of predicted mesocarnivore occurrence across the study area in summer and winter for the first and last years of the study (green and blue panels, respectively). Gray panel maps represent the winter and summer predicted stable state occurrence ($\varphi^{\mathrm{eq}}$).

**FIGURE 6:**
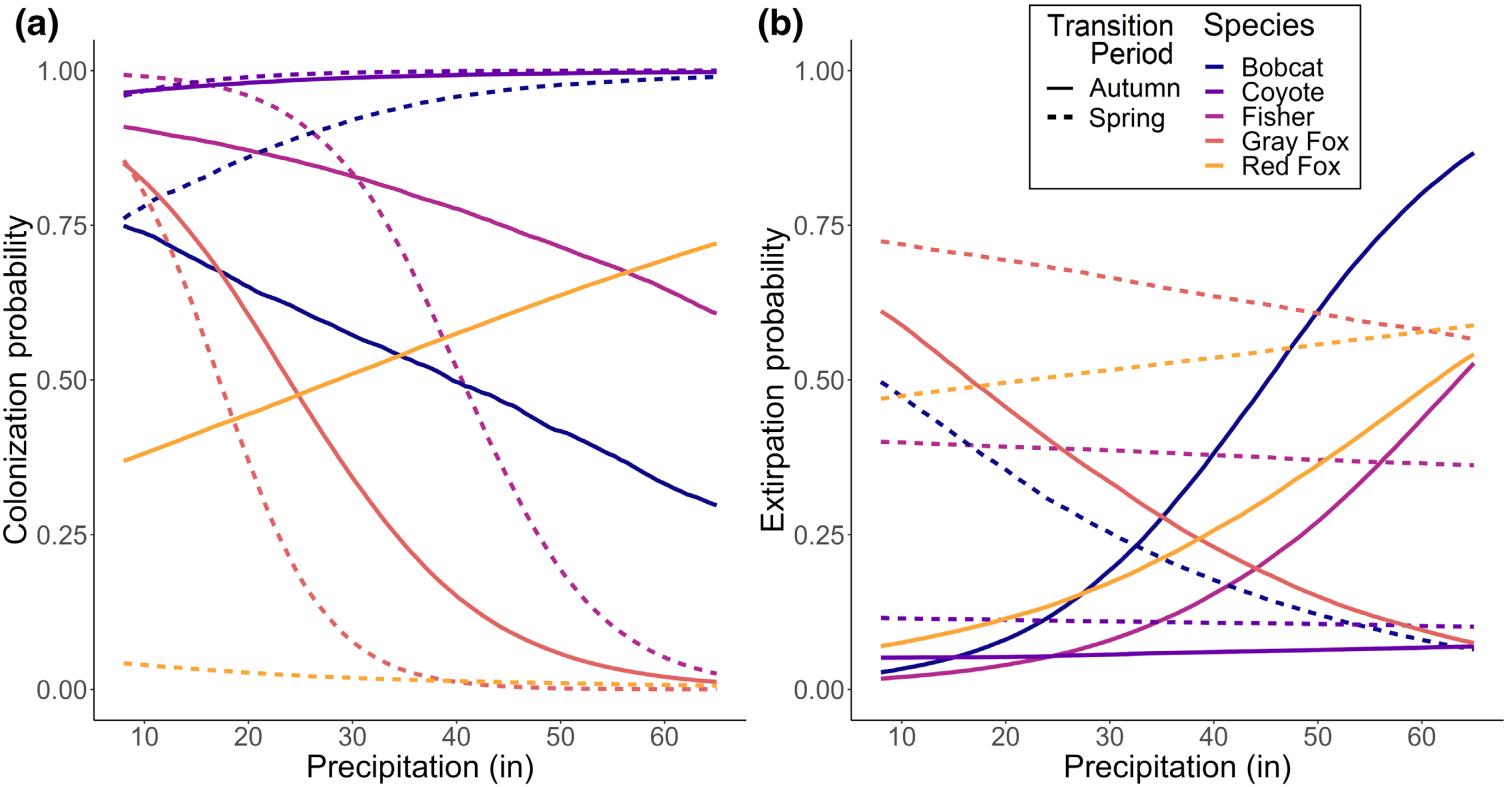
Relationship of colonization (a) and extirpation (b) probabilities to changes in seasonal precipitation in different transition periods for mesocarnivore species. Dashed lines represent the predicted posterior median for the effect of winter snow on the spring transition period, while solid lines represent the predicted posterior median for the effect of summer rain on the autumn transition period.

Bobcat occupancy dynamics were most impacted by climatic variables (Appendix S2: Table S1). Occupancy was higher in summer than winter (Figure 2), and bobcat had higher γ in winter than summer (*β*_season_ = 1.63; Figure 4a). Our models indicated that bobcat occupancy estimates increased in winter (*c*_w_ = 0.02), and summer (*c*_s_ = 0.34) from 2019 to 2023 (Figure 2). The only season when bobcat occupancy declined was in summer of 2020 ($\bar{l}=0.84$, Figure 2). Predictions of occurrence across the state showed that bobcat summer occurrence increased from first to last year, particularly in the northwestern region of the state (Figure 5; Appendix S2: Figure S6). Summer occurrence in 2022 was also trending higher than occupancy probabilities in the summer stable state, whereas winter occurrence in 2023 was trending lower than the winter stable state (Figure 5). Occupancy dynamics were associated with seasonal precipitation showing that sites with more rain in summer and less snow in winter were more likely to become colonized by bobcat in the following season (*β*_season_ = 1.63, *β*_precip:season_ = 1.27; Figure 6a).

**FIGURE 7:**
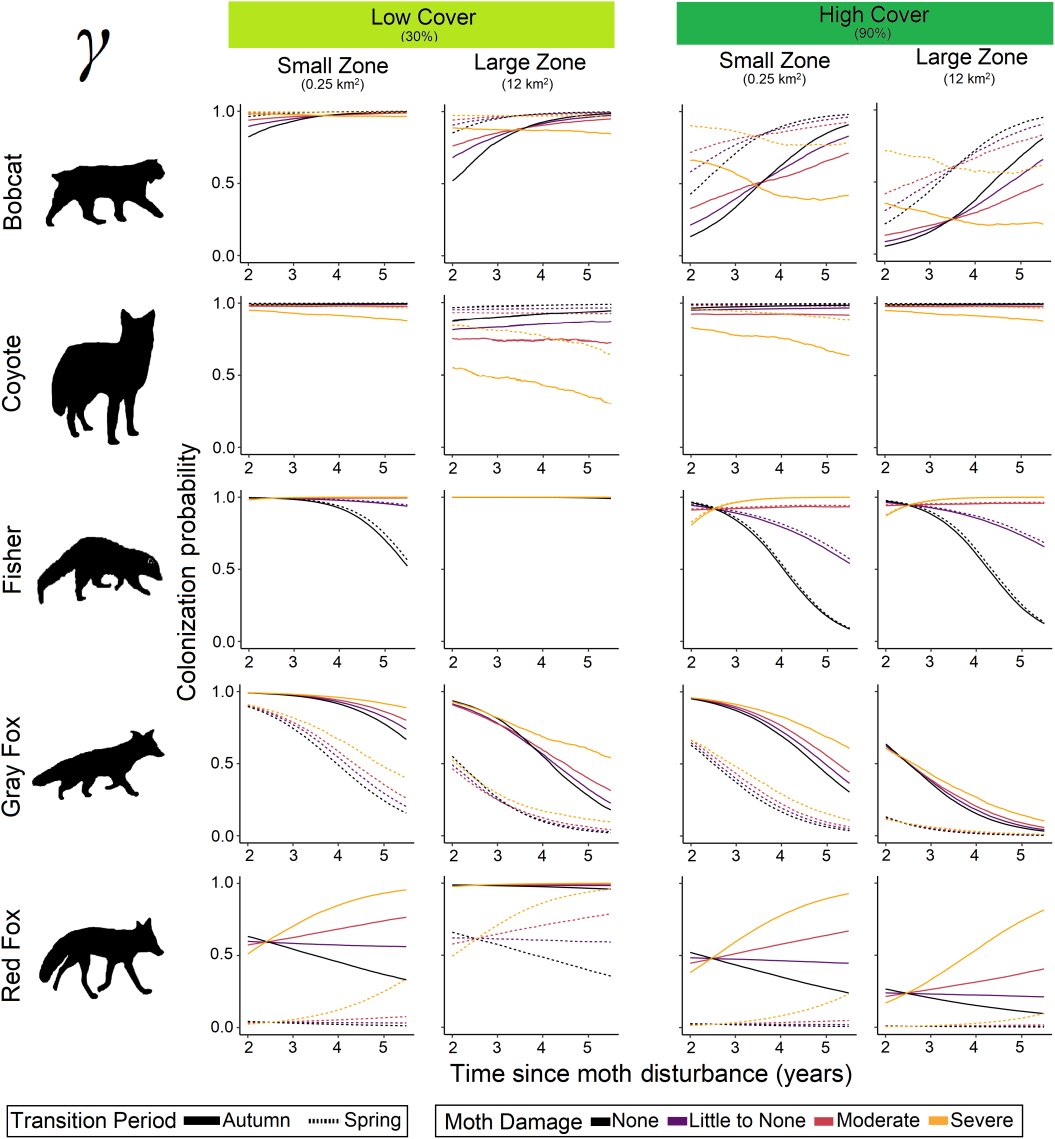
Colonization probability responses of all mesocarnivores to changes in cover, zone size, transition period, moth damage, and time since disturbance. Each line represents the predicted posterior median.

**FIGURE 8:**
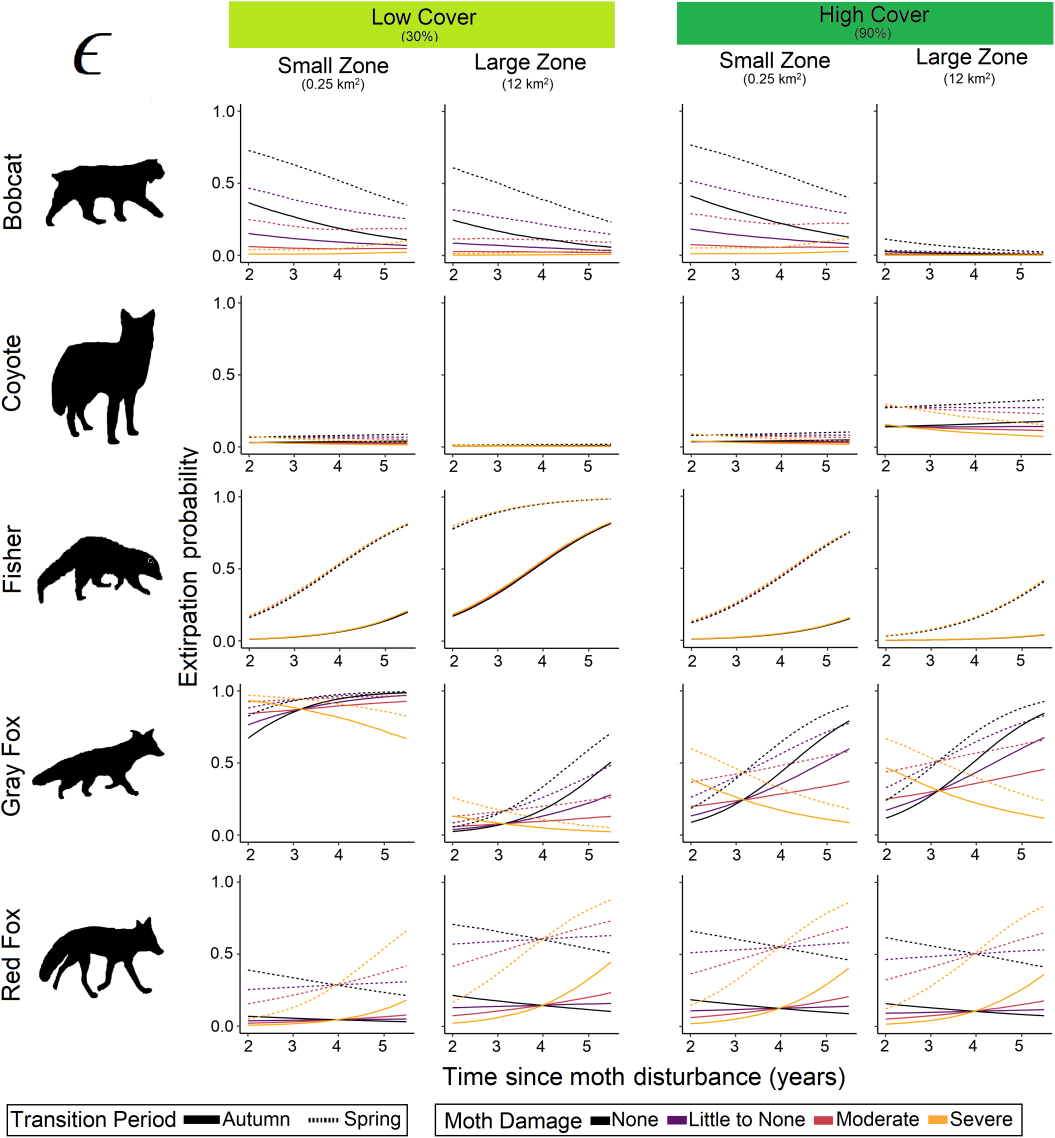
Extirpation probability responses of all mesocarnivores to changes in cover, zone size, transition period, moth damage, and time since disturbance. Each line represents the predicted posterior median.

In response to anthropogenic disturbance, bobcat were most likely to initially occupy sites far from roads (*β*_road_dist_ = 1.14; Figure 3a) and detection probabilities were higher in areas of low cover (*β*_cover_ = −0.06). Colonization and extirpation probabilities were associated with zone size and cover and indicated that bobcats were more likely to colonize areas with low cover (*β*_cover_ = −1.07; Figure 7, Appendix S2: Figure S1) and more likely to extirpate small zones and any zone with low cover (*β*_zone_area_ = −1.18, *β*_cover_ = −0.32, *β*_zone_area:cover_ = −0.36; Figure 8). In regard to natural disturbance, bobcat were more likely to initially occupy sites with little moth damage (*β*_moth_ = −0.76; Figure 3a) and colonization probabilities were associated with forest succession (i.e., increasing TSD; *β*_TSD_ = 1.14; Figure 4a). However, this response varied with moth damage severity where bobcat were more likely to colonize sites as time passed in areas with up to moderate moth damage, but when moth damage became severe, bobcats became less likely to colonize those areas (Figure 7).

#### 3.2.2 Coyote

**FIGURE 9:**
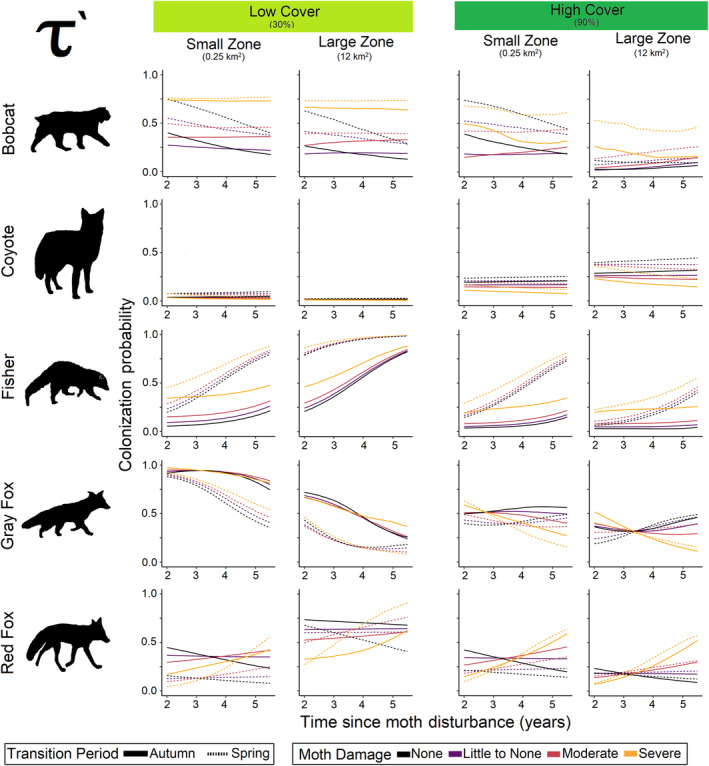
Turnover probability responses of all mesocarnivores to changes in cover, zone size, transition period, moth damage, and time since disturbance. Each line represents the predicted posterior median.

Coyote occupancy was associated with anthropogenic disturbance in the form of available cover within a zone with lower extirpation in small zones and zones of low cover (*β*_zone_area_ = −1.18, *β*_cover_ = −0.32, *β*_zone_area:cover_ = −0.36; Figure 8), and higher *p* in low cover zones (*β*_cover_ = −0.32; Figure 3b). Additionally, coyote occupancy dynamics were not associated with natural disturbance (Figures 4, 7, 8 and 9, Appendix S2: Figure S2) and occupancy remained stable from 2019 to 2023 (Figure 9).

#### 3.2.3 Fisher

Fisher occupancy dynamics were most associated with seasonal precipitation, followed by natural disturbance and cover (Figures 3 and 4). Fisher occupancy declined in both seasons at different rates (*c*_s_ = 0.98, *c*_w_ = 1.00, Figure 2) from 2019 to 2023 with larger declines in summer (*β*_season_ = 2.49; Figure 4b). Fisher were less likely to colonize a site that received high precipitation in the previous season (*β*_precip_ = −0.42, *β*_precip:season_ = −1.54; Figure 6a), however they became more likely to leave sites with more precipitation only in the autumn transition period in response to previous summer rain (*β*_precip_ = 0.93, *β*_season_ = 2.49, *β*_precip:season_ = −0.97; Figure 6b). Predictions of occurrence across the state showed that fisher summer occurrence declined from first to last year, particularly in the northwestern and coastal regions of the state, and summer occurrence in 2022 was trending lower than occupancy probabilities in the predicted summer stable state (Figure 5; Appendix S2: Figure S7).

Our models indicated that fisher had higher turnover rates in areas of low cover and the lowest turnover rates in large zones of high cover (Figure 9). Extirpation probability was largely associated with anthropogenic disturbance as fisher were more likely to leave low cover areas and the least likely to leave large zones of high cover (*β*_zone_area_ = −0.10, *β*_cover_ = −0.77, *β*_zone_area:cover_ = −0.64; Figure 8). In regard to natural disturbance, fisher were less likely to initially occupy sites with higher moth damage (*β*_moth_ = −0.46), our models showed declines in γ in areas with little or no moth damage over time, whereas in moderate to severely damage areas γ remained high (*β*_moth_ = 0.98, *β*_TSD_ = −1.24, *β*_moth:TSD_ = 1.00; Figure 7; Appendix S2: Figure S3). Additionally, fisher had higher site turnover as time passed (Figure 9).

#### 3.2.4 Gray fox

Gray fox site‐level occupancy estimates severely declined in both seasons from the beginning to end of the study (*c*_s_ = 0.99, *c*_w_ = 0.87; Figure 2), however, the only instance when gray fox occupancy increased occurred between the summer of 2019 and 2020 where increases occurred in 87% of the study area ($\bar{l}=1.16$, Appendix S2: Figure S8). Gray fox appear spatially to be trending toward the stable state distribution in winter only when comparing the predicted occurrence across the state with the predicted stable state, and in 2023 mean summer occupancy was 0.06 (Figure 5). Occupancy dynamics were largely associated with season, with lower γ (*β*_season_ = −1.34; Figure 4a) and higher *ϵ* (*β*_season_ = 1.16; Figure 4b) in the spring transition period. Precipitation was also largely associated with gray fox occurrence with higher γ at sites with less precipitation (*β*_precip_ = −1.43; Figure 6a), but opposite and less strong association occurred with *ϵ* where gray fox were more likely to leave sites with less precipitation (*β*_precip_ = −0.66; Figure 6b).

Regarding anthropogenic disturbance, gray fox were the only species with a large response to zone size and were less likely to colonize larger zones (*β*_zone_area_ = −1.04, Figure 7), and were less likely to extirpate larger zones with more cover (*β*_zone_area_ = −0.49, *β*_cover_ = −0.24, *β*_zone_area:cover_ = 0.70; Figure 8). Site turnover was highest in small zones of low cover and during the spring (Figure 9). While there were no associations of initial occupancy with moth damaged areas (Figure 3a), gray fox γ was negatively associated with natural disturbance in the form of forest succession (*β*_TSD_ = −1.34, Figure 7), regardless of moth damage severity. However, *ϵ* was largely associated with natural disturbance and the likelihood that gray fox left a site was highest in small zones of low cover (*β*_zone_area_ = 0.10, *β*_cover_ = 0.14, *β*_zone_area:cover_ = −0.21; Figure 8). Extirpation probability increased over time since moth outbreak and as moth damage severity increased (*β*_moth_ = −0.22, *β*_TSD_ = 0.97) with the exception of areas with severe moth damage where gray fox became less likely to leave a site (*β*_moth:TSD_ = −0.45; Figure 8).

#### 3.2.5 Red fox

Our models indicated declines in red fox occupancy in both seasons from 2019 to 2023 (*c*_s_ = 0.99, *c*_w_ = 0.99; Figure 2). Our model predictions of occupancy across the state indicated that red fox occurrence remained stable between summer and winter as a result of alternating high γ (*β*_season_ = −3.85; Figure 7) and low *ϵ* (*β*_season_ = 1.91; Figure 8) between seasons, however, there appeared to be annual variation in occurrence (Figure 5; Appendix S2: Figure S9). Despite declines in occupancy, red fox occurrence appears to be spatially trending toward the stable state distribution, however mean occupancy in the final seasons was low at 0.09 and 0.08, respectively (Figure 5). Additionally, occupancy dynamics were largely associated with summer rain, where red fox were more likely to colonize sites during the autumn transition period that had high rainfall in the preceding summer (*β*_precip_ = 0.33, *β*_precip:season_ = −0.81; Figure 6a) and they became more likely to leave those same sites, but at a lower rate than colonization (*β*_precip_ = 0.64, *β*_precip:season_ = −0.54; Figure 6b).

Regarding anthropogenic disturbance, red fox initial occupancy was associated with distance to road (*β*_road_dist_ = 0.21, p_pos = 0.78; Figure 3a). Colonization (*β*_cover_ = −0.92, *β*_zone_area:cover_ = −0.71) and extirpation probability (*β*_zone_area_ = 0.10, *β*_zone_area:cover_ = −0.21) associations with zone size and cover only were of small magnitudes in small to medium sized zones, however, in large zones as cover increased, turnover probability declined with the lowest turnover in large zones of high cover (Figure 9, Appendix S2: Figure S5). Initial occupancy was associated with natural disturbance as red fox were likely to occupy sites with higher moth damage (*β*_moth_ = 0.34; Figure 3a). Colonization was associated with forest succession (i.e., TSD increased; *β*_TSD_ = −1.34; Figure 4a) and this response was modified by moth damage severity where γ in moderate to severely damaged areas increased as time passed (*β*_moth_ = 0.35, *β*_moth:TSD_ = 0.35; Figure 7). Red fox also became more likely to extirpate sites with moth damage and less likely to extirpate sites without moth damage over time (*β*_moth:TSD_ = 0.35; Figure 8).

## 4 DISCUSSION

We found all mesocarnivore species except coyote have declined in occurrence between 2019 and 2023 in at least one season, and showed moderate‐to‐strong support for effects of anthropogenic disturbance, natural disturbance, and climate on occupancy dynamics. Our results support the need to simultaneously examine the impacts of both disturbance types as understanding species' responses to changes on the landscape is context dependent. The seasonal difference between winter and summer generally had the largest effects across all species' colonization and extirpation. The influence of seasonal precipitation, however, only had large effects on bobcat extirpation, and overall fisher and gray fox occupancy dynamics. Anthropogenic disturbance in the form of roadless zones and cover availability within zones had strongly supported large negative impacts on fisher, gray fox, and red fox colonization (Figure 5; Table S2). Additionally, bobcat and fisher were less likely to occupy smaller areas with less cover (Appendix S2). Coyote responded positively to anthropogenic disturbance, having lower extirpation in smaller, more exposed zones. The impacts of natural disturbance from the spongy moth defoliation event had large effects on fisher and gray fox occupancy dynamics, and smaller effects on red fox dynamics and bobcat extirpation. For all species but coyote, all three variable categories impacted occupancy dynamics and as such we considered the combined effects for each species in detail to best understand each species distribution response.

### 4.1 Bobcat

Bobcat occurrence appeared to be stabilizing across the state with an overall increase in occupancy from 2019 to 2023. While we predicted bobcat occupancy would be higher in winter associated with larger winter home ranges and movements (Lovallo & Anderson, 1996; McNit et al., 2020), our models indicated that bobcat occupancy in Rhode Island was actually higher in summer than winter. This may be related to the elusiveness of the species and difficulty detecting bobcat on trail cameras. With very low detection rates of bobcats in our study, we may have had higher occupancy in summer related to increased probability of detection as the species, particularly females, are not ranging as widely as in winter and have higher site fidelity in summer related to denning locations and sources of reliable prey (Litvaitis et al., 1986; Lovallo & Anderson, 1996). Interestingly, the only season where bobcat occupancy declined was from the summer of 2019 to 2020. During the summer where the increase was observed in 2020, work‐from‐home orders and other travel restrictions were being enforced due to the coronavirus pandemic resulting in lower traffic volumes. As bobcats have been known to avoid roads, we speculate that movements may have expanded during this summer season in response to decreased traffic volume resulting in similar movement to winter. Additionally, we thought that colonization probabilities would be lower in areas with more snow as bobcats are known to shrink their movements in deep snow (McCord, 1974), however, our models indicated the opposite. While bobcat movement distances shrink in deep snow, they also shift their movements to trails which are prevalent across the state in the form of roads and hiking trails (McCord, 1974). Our findings indicate that snow may not limit bobcat distributions in Rhode Island.

Our models indicated that bobcat initial occupancy was highest in areas far from roads, providing evidence that bobcat avoided roads (Mayer et al., 2022). However, we also predicted that bobcat would have higher colonization in medium to larger zones with low cover representative of areas with space to stay away from roads and with potentially early successional habitat, but our models indicated that bobcat had higher colonization in small zones of low cover. This may be related to the transient nature of the species as mentioned previously, as we also saw the highest extirpation rates in small areas with low cover, indicating bobcat are utilizing small zones between roads to move across the state but do not use those areas year‐round. We did find support that bobcat are using large areas with high cover year‐round and that those areas are important for maintaining bobcat populations in the state.

We predicted that bobcat would be more likely to colonize severely moth damaged areas over time because the species' capitalizes on early successional habitat that is created from natural disturbance (Fuller & DeStefano, 2003) which was supported by our models. Initially, bobcat occupied areas with little moth damage, but in the first 2–3 years post‐disturbance they began moving into severely moth damaged areas. After year 3.5, colonization probability declined, however, extirpation was very low in severe moth damaged areas throughout the study suggesting bobcat moved into severely moth damaged areas during early succession and stayed in those areas. We infer that areas of natural disturbance may be providing bobcats with abundant prey opportunities suited for their hunting style and the areas of cover may be providing adequate denning habitat to meet their needs (McNit et al., 2020).

Lastly, bobcat winter occurrence in Rhode Island appears to be trending toward the stable state (Figure 5). If all conditions remain stable and the forest remains in the current successional state, bobcat occupancy would be expected to decrease slightly in summer and increase slightly in winter. Additionally, future low‐emission climate projections predict an increase in both summer rainfall and winter snow in this region (Collins et al., 2013). As increased summer precipitation is likely to reduce bobcat colonization and increase extirpation in the autumn transition period, we may expect a decline in occurrence in winter. However, we found bobcat responded positively to winter snow, so as snowfall increases across the study region in the future, we may see declines in spring extirpation and increases in colonization which may balance out bobcat occurrence throughout the state (Figure 6).

### 4.2 Coyote

Coyote were the only species that did not respond to the natural disturbances (i.e., no support for effects of moth damage or time since disturbance); however, colonization and extirpation did respond positively to anthropogenic effects related to zone size and available cover supporting our predictions (Appendix S2). We were not surprised to find that coyotes are well adapted to anthropogenic effects in Rhode Island as there is ample support in the literature documenting coyote becoming widespread across various landscape configurations (Breck et al., 2019; Gompper, 2002; Hinton et al., 2015). Our findings supported our predictions that coyote occurrence would be widespread and stable across the state. With low extirpation and high colonization probabilities coyotes will most likely remain widespread across Rhode Island regardless of climate scenarios and changes in disturbance.

### 4.3 Fisher

As a species that is well known to require forests with high canopy‐cover, it was not surprising to find that fisher had the lowest turnover in large, high cover zones and were less likely to leave areas with high cover than anywhere else, supporting our predictions and suggesting various types of cover are important for the species (Kelly, 1978; Zielinski et al., 2004; Lofroth et al., 2010; Figure 9; Figure S2). The seasonal dynamics and occupancy of low cover zones during the winter season supports recent evidence that the eastern fisher population is capable of tolerating some level of anthropogenic disturbance (Brown et al., 2012; Naney et al., 2012; Raley et al., 2012).

Contrary to our prediction that moth damaged areas would not provide the cover fisher require, colonization was highest in moderate to severely moth damaged areas and fisher became less likely to colonize areas with no moth damage over time (Appendix S2). If fisher populations are declining as suggested by declines in occurrence (Figure 5), our results may show summer fisher home ranges concentrated around large, high cover zones with no moth damage as these areas had the lowest turnover (i.e., most stability in occupancy). However, our results also indicate that fisher still utilized other surrounding areas with moth damage as needed during the winter season, indicating that fisher benefit from moth damaged areas but those areas are no longer sufficient for fisher in early successional phase to remain occupied year‐round.

Our predictions of seasonal variation in occupancy and that fisher would respond negatively to snow were both supported. Fisher were most likely to occupy sites that received less than 30 inches of snow, but at seasonal snowfall totals above 40 inches, fisher were most likely to leave those sites. While our models only show responses to seasonal snowfall totals and do not account for snow density which has also been known to impact fisher associations with snow, our findings support evidence from previous studies that fisher avoid areas with more snow (Powell, 1993; Powell & Zielinski, 2003). Lastly, fisher occupancy declined across Rhode Island over the course of the study (Figures 2 and 5). Fisher summer occupancy in 2022 was below the predicted summer stable state (Figure 5), suggesting that if all variables were to remain stagnant fisher occupancy should increase slightly in summer. However, with the drastic decline in occupancy from 2019 to 2023 and our findings that fisher respond negatively to both increases in summer rain and winter snow (Figure 6), this is a major concern for fisher populations in the future under both emissions scenarios that predict increases in seasonal precipitation in this region.

### 4.4 Gray fox

In the first year of the study, occupancy was initially low across the study area with gray fox concentrating in areas with more cover, as we expected for a disturbance‐sensitive, forest‐dependent species living in a highly disturbed landscape (Hall, 1981). Additionally, our models showed that high cover areas had the most stability in occupancy which supported our predictions that gray fox would be sensitive to habitat loss and road disturbance (Cypher, 2003). However, we found evidence that gray fox exhibited some level of plasticity in their response to anthropogenic disturbances where they occupied areas with low to moderate cover in the winter (Appendix S2). Large, low cover zones may contain agricultural lands or large bodies of water, so there may be potential for gray fox to be using areas of edge habitat around fields that were not specified in this analysis (Follman, 1973; Wood, 1958). Previous studies have shown that when gray fox use mixed agricultural lands they require adjacent tree cover, and those living in areas with anthropogenic disturbance maintain core home ranges within natural areas (Riley, 2006). Interestingly, the only observed increase in gray fox occupancy was from the summer of 2019 to 2020, the same summer when bobcat occurrence declined during the coronavirus pandemic. At this time, traffic volume was low and perhaps this facilitated gray fox movement and allowed the species to move more comfortably across the landscape, thus occupying new territories. Our findings support the ability of gray foxes to benefit from anthropogenic disturbance (Bateman & Fleming, 2012; Harrison, 1997; Riley, 2006), but also emphasize that large areas of cover with little human influence are crucial for this species.

Additionally, we found that the effects of anthropogenic disturbance on gray fox occupancy dynamics were compounded with effects of natural disturbance (Figure 9; Appendix S2). We predicted that gray fox would require undamaged forests and would not occupy areas with moth damage, which was partially supported. Interestingly, gray fox were more likely to leave areas with moderate moth damage or less but were less likely to leave areas with severe moth damage. The larger the zone size, more severe the moth damage, and less cover, the more likely gray fox would colonize a site and it would remain occupied (Figure 5; Appendix S2). It is unclear whether this shift in occurrence to more fragmented and exposed areas is due to lack of resources for gray fox in forested areas, creation of more desirable habitat from disturbance, or because of potential competition with fisher that may be concentrating in large zones of high forest cover or suppression by coyote (Smith et al., 2018).

We observed a large decline in gray fox occupancy over the course of the study, however, predicted occupancy patterns appear to be trending toward the stable state in winter (Figures 2 and 5). Similar to fisher, summer occurrence in 2022 was well below the stable summer state, indicating potential for gray fox occupancy to increase, however, given their sensitivity to changes in the forest as a result of the moth damage caution should be made when assuming the future of population stability without continued monitoring. We also must take into consideration our finding of negative responses of gray fox to increased seasonal precipitation (Figure 6). Our prediction that gray fox would respond positively to summer rain as it is associated with prey densities (Meserve et al., 2011) was partially supported where gray fox were more likely to colonize than extirpate sites when rainfall was below 30″, however above 30″ they became slightly more likely to leave sites than colonize. With these responses, we may expect gray fox occupancy to decline below the stable state if climate projections are accurate. However, it also appeared that gray fox exhibited plasticity in their responses to changing forest structure as areas with severe outbreak enter early succession. Continued monitoring of gray fox occurrence would aid in identifying potential shifts in occupancy dynamics as the species may respond differently to increasing stages of forest regeneration.

### 4.5 Red fox

We had mixed support for our predictions for red fox response to anthropogenic disturbance were supported. We predicted red fox would occupy areas near roads, but our models indicated that red fox were more likely to be found far from roads initially. Red fox are known to benefit from edge habitat near roads and they hunt in other edge habitat that would be related to low cover areas where we also expected occupancy to be more stable (Ruiz‐Capillas et al., 2021). Occupancy dynamics related to anthropogenic disturbance were mediated by seasonal variation, where shifts in distributions related to zone size and cover occurred primarily in the autumn transition period. If a red fox occupied a large zone of low cover in the summer, they remained there throughout winter and the following summer. Additionally, if a large zone of low cover was unoccupied in summer, there was a high likelihood that the site became occupied in the winter and remained occupied through the next summer, supporting our predictions that red fox benefit from anthropogenic disturbance.

Regarding natural disturbance, our results had mixed support for our predictions that red fox would respond positively to moth damage as responses varied in conjunction with season, cover and zone size. Red fox moved into areas with severe moth damage in autumn and were likely to stay in those areas through winter and into the next summer unless they were areas of high cover. In high cover areas with severe damage, red fox were more likely to leave in spring for large, low cover zone as time passed. So, as succession progressed red fox left large high cover areas that had been severely naturally disturbed for areas with high human disturbance in summer. These findings suggest that when moth damage occurs in already fragmented habitats, this benefits red fox, and that there may be a temporal threshold of forest regeneration in expansively disturbed areas after which the disturbance is no longer beneficial to the species.

Like fisher and gray fox, we observed declines in red fox occurrence across Rhode Island, and it appeared that red fox occupancy patterns are very near the stable state in both seasons (Figures 2 and 5). Our findings of spring transition periods to be most influential in the following year's occurrence and significant state‐wide declines in red fox occurrence may be indicative of declines in population densities. It is notable that while red fox are known to be suppressed by coyote, in areas where coyote occurrence was lowest we did not see positive responses of red fox. Red fox occurrence in the future is predicted to respond positively to increased summer precipitation, but there is little response to increased snowfall, suggesting if prey densities do increase in response to high seasonal rainfall red fox occupancy may increase in the future (Cypher, 2003; Meserve et al., 2011).

## 5 CONCLUSION

We found gray fox and fisher to be highly sensitive to disturbance with large responses associated with both disturbance types and climate. Bobcat had large responses associated with climatic conditions, red fox had large responses associated with season and forest succession, and coyote occurrence was positively associated with anthropogenic disturbance. Both fox species and coyote showed plasticity in their responses to rapid environmental changes caused by disturbance, suggesting their ability to adapt to changing conditions of a similar magnitude in the future. However, the persistence of these species in Rhode Island with increasing forest succession and under more severe environmental change is uncertain. While gray fox exhibit plasticity in their responses, negative impacts of increased precipitation in the future may exceed the limitations of their ability to acclimatize to changing conditions. Our findings indicate fisher and gray fox occurrence was below what we would expect to see if occupancy was stable, suggesting potential for increases in populations in the coming years if the state of the natural landscape and climate conditions were to remain stagnant from the last year of the study. While bobcat and red fox were predicted to respond positively to future climate scenarios, fisher and gray fox were not. Large contiguous zones of cover were beneficial to fisher and gray fox and there is potential for both species to respond positively to naturally disturbed areas as those forests enter the late successional phase. Our study only captures the responses of mesocarnivores to the first 6 years of succession post moth damage and making inference on mesocarnivore responses to later successional phases would be speculative. Large contiguous zones of cover were beneficial to most mesocarnivores species, thus we emphasize the importance of conserving large tracts of land or increasing connectivity between contiguous areas of cover in this region. Here we provide insight into the initial responses of mesocarnivores to large‐scale natural and anthropogenic disturbance and climate conditions, and continued monitoring would allow even further understanding of these dynamics as forest succession continues and these species experience wider variability in climatic conditions.

## AUTHOR CONTRIBUTIONS

**Laken S. Ganoe:** Conceptualization (lead); data curation (equal); formal analysis (lead); investigation (equal); methodology (equal); visualization (lead); writing – original draft (lead). **Amy E. Mayer:** Data curation (equal); investigation (equal); methodology (equal); writing – review and editing (equal). **Charles Brown:** Funding acquisition (supporting); methodology (equal); supervision (supporting); writing – review and editing (equal). **Brian D. Gerber:** Conceptualization (supporting); formal analysis (supporting); funding acquisition (lead); methodology (equal); supervision (lead); visualization (supporting); writing – original draft (supporting); writing – review and editing (equal).

## CONFLICT OF INTEREST STATEMENT

There are no conflicts of interest to declare.

## Supporting information


Appendix S1–S2

## Data Availability

Datasets and code for analyses available at https://zenodo.org/doi/10.5281/zenodo.10459833.
