# Supplementary material for: Mesocarnivore sensitivity to natural and anthropogenic disturbance leads to declines in occurrence and concern for species persistence
Source: Ecol Evol. 2024 Jul 21;14(7):e70043. doi: 10.1002/ece3.70043 (PMC11260557; doi:10.1002/ece3.70043)
Supplement: Supplementary file 1 — Appendix S1–S2 [file ECE3-14-e70043-s001.docx]

**Appendix 1**

Methods Appendix


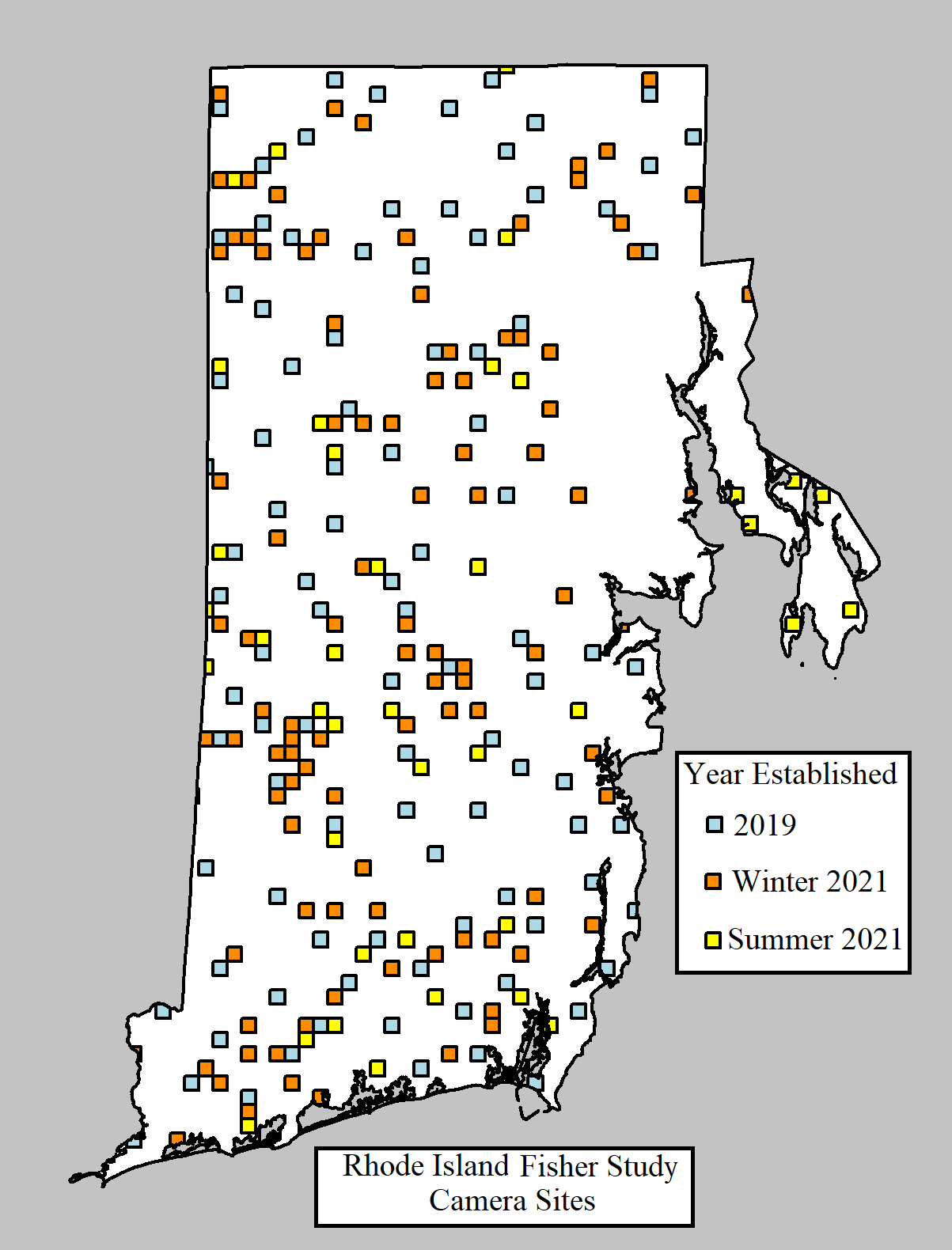


**Figure S1 -** Map depicting addition of survey sites in increasing years of survey. 100 sites set forth by Mayer et al. 2022 depicted in blue. Sample size was increased by 100 additional sites in winter of 2021 (orange), and then again in the following summer by an additional 40 sites.

Data censorship: Fisher, coyote, gray fox, and red fox are harvested in the state, however fisher are the only species that regularly have more than 20 individuals harvested. Fisher detection data was censored during fisher trapping season from December 1 – 24 of each winter season. There is not a harvest season for bobcat in Rhode Island.

**Appendix 2**

Results Appendix

**Table S1 -** Posterior median coefficients for all variables by species. Anthropogenic disturbance variables are highlighted in orange, natural disturbance in yellow, and climatic variables in blue. The strength of support is indicated by *p_pos* values where values between 0 – 0.1 and 0.9 – 1 are considered strong negative and positive support, respectively. Values between 0.1 – 0.3 and 0.7 – 0.9 are considered moderate negative and positive support, respectively. Values in bold indicate supported terms.

|  | **Bobcat** | | **Coyote** | | **Fisher** | | **Gray Fox** | | **Red Fox** | |
| --- | --- | --- | --- | --- | --- | --- | --- | --- | --- | --- |
| $\boldsymbol{\psi}_{\boldsymbol{1}}$ | β | *p_pos* | β | *p_pos* | β | *p_pos* | β | *p_pos* | β | *p_pos* |
| β_0_ | **2.19** | ***1.00*** | **2.70** | ***1.00*** | **3.24** | ***1.00*** | -0.15 | *0.31* | **-0.56** | ***0.01*** |
| β_1_ (cover) | -0.01 | *0.54* | **-1.00** | ***0.01*** | 0.09 | *0.62* | **0.43** | ***0.90*** | -0.05 | *0.41* |
| β_2_ (road_dist) | **1.14** | ***0.91*** | **0.46** | ***0.80*** | 0.46 | *0.68* | **-0.35** | ***0.15*** | **0.21** | ***0.78*** |
| β_3_ (moth) | **-0.76** | ***0.15*** | 0.20 | *0.69* | **-0.46** | ***0.20*** | 0.13 | *0.64* | **0.34** | ***0.94*** |
| ***p*** |  |  |  |  |  |  |  |  |  |  |
| β_0_ | **-3.08** | ***0.00*** | **-0.81** | ***0.00*** | **-1.03** | ***0.00*** | **-3.41** | ***0.00*** | **-1.67** | ***0.00*** |
| β_1_ (zone_area) | 0.04 | *0.66* | **0.15** | ***0.99*** | **-0.14** | ***0.02*** | **0.44** | ***0.95*** | **-0.22** | ***0.05*** |
| β_2_ (cover) | **-0.06** | ***0.28*** | **-0.08** | ***0.07*** | **0.10** | ***0.92*** | **-0.16** | ***0.23*** | **0.11** | ***0.81*** |
| σ (station) | **1.09** | ***1.00*** | **0.68** | ***1.00*** | **0.82** | ***1.00*** | **2.09** | ***1.00*** | **1.21** | ***1.00*** |
| $\boldsymbol{\gamma}$ |  |  |  |  |  |  |  |  |  |  |
| β_0_ | 0.57 | *0.65* | **3.91** | ***1.00*** | **2.63** | ***1.00*** | 0.12 | *0.58* | **0.18** | ***0.74*** |
| β_1_ (precip) | -0.39 | *0.37* | 0.59 | *0.67* | **-0.42** | ***0.29*** | **-1.43** | ***0.03*** | **0.33** | ***0.85*** |
| β_2_ (season) | **1.63** | ***0.84*** | 0.88 | *0.69* | 0.71 | *0.68* | **-1.34** | ***0.10*** | **-3.85** | ***0.00*** |
| β_3_ (precip:season) | **1.27** | ***0.83*** | **0.88** | ***0.72*** | **-1.54** | ***0.08*** | **-1.25** | ***0.16*** | **-0.81** | ***0.13*** |
| β_4_ (zone_area) | -0.60 | *0.31* | **0.47** | ***0.71*** | **0.76** | ***0.86*** | **-1.04** | ***0.08*** | 0.13 | *0.68* |
| β_5_ (cover) | **-1.07** | ***0.17*** | **0.73** | ***0.73*** | **-1.49** | ***0.02*** | **-0.75** | ***0.21*** | **-0.92** | ***0.00*** |
| β_6_ (zone_area:cover) | 0.17 | *0.53* | 0.19 | *0.55* | -0.62 | *0.25* | -0.20 | *0.47* | **-0.71** | ***0.04*** |
| β_7_ (moth) | -0.04 | *0.44* | **-0.56** | ***0.29*** | **0.98** | ***0.91*** | 0.15 | *0.63* | **0.35** | ***0.95*** |
| β_8_ (TSD) | **1.14** | ***0.81*** | 0.16 | *0.57* | **-1.24** | ***0.07*** | **-1.34** | ***0.00*** | **-0.17** | ***0.25*** |
| β_9_ (moth:TSD) | -0.36 | *0.37* | -0.16 | *0.43* | **1.00** | ***0.86*** | 0.08 | *0.58* | **0.35** | ***0.90*** |
| $\boldsymbol{\epsilon}$ |  |  |  |  |  |  |  |  |  |  |
| β_0_ | **-2.76** | ***0.00*** | **-3.01** | ***0.00*** | **-2.93** | ***0.00*** | **-0.57** | ***0.11*** | **-1.95** | ***0.00*** |
| β_1_ (precip) | **1.20** | ***0.91*** | 0.08 | *0.54* | **0.93** | ***0.92*** | **-0.66** | ***0.15*** | **0.64** | ***0.83*** |
| β_2_ (season) | **1.20** | ***0.91*** | **0.82** | ***0.97*** | **2.49** | ***1.00*** | **1.16** | ***0.98*** | **1.91** | ***1.00*** |
| β_3_ (precip:season) | **-1.84** | ***0.02*** | -0.11 | *0.43* | **-0.97** | ***0.08*** | **0.49** | ***0.76*** | **-0.54** | ***0.22*** |
| β_4_ (zone_area) | **-1.18** | ***0.09*** | **0.27** | ***0.94*** | **-0.10** | ***0.28*** | **-0.49** | ***0.04*** | **0.10** | ***0.71*** |
| β_5_ (cover) | **-0.32** | ***0.30*** | **0.54** | ***0.99*** | **-0.77** | ***0.00*** | **-0.24** | ***0.26*** | 0.14 | *0.69* |
| β_6_ (zone_area:cover) | **-0.36** | ***0.28*** | **0.44** | ***0.96*** | **-0.64** | ***0.00*** | **0.70** | ***0.98*** | **-0.21** | ***0.21*** |
| β_7_ (moth) | **-0.81** | ***0.06*** | -0.12 | *0.32* | 0.01 | *0.54* | **-0.22** | ***0.20*** | -0.06 | *0.38* |
| β_8_ (TSD) | **-0.38** | ***0.29*** | 0.03 | *0.55* | **1.09** | ***1.00*** | **0.97** | ***0.99*** | -0.01 | *0.48* |
| β_9_ (moth:TSD) | 0.25 | *0.64* | -0.09 | *0.37* | 0.00 | *0.50* | **-0.45** | ***0.10*** | **0.35** | ***0.92*** |


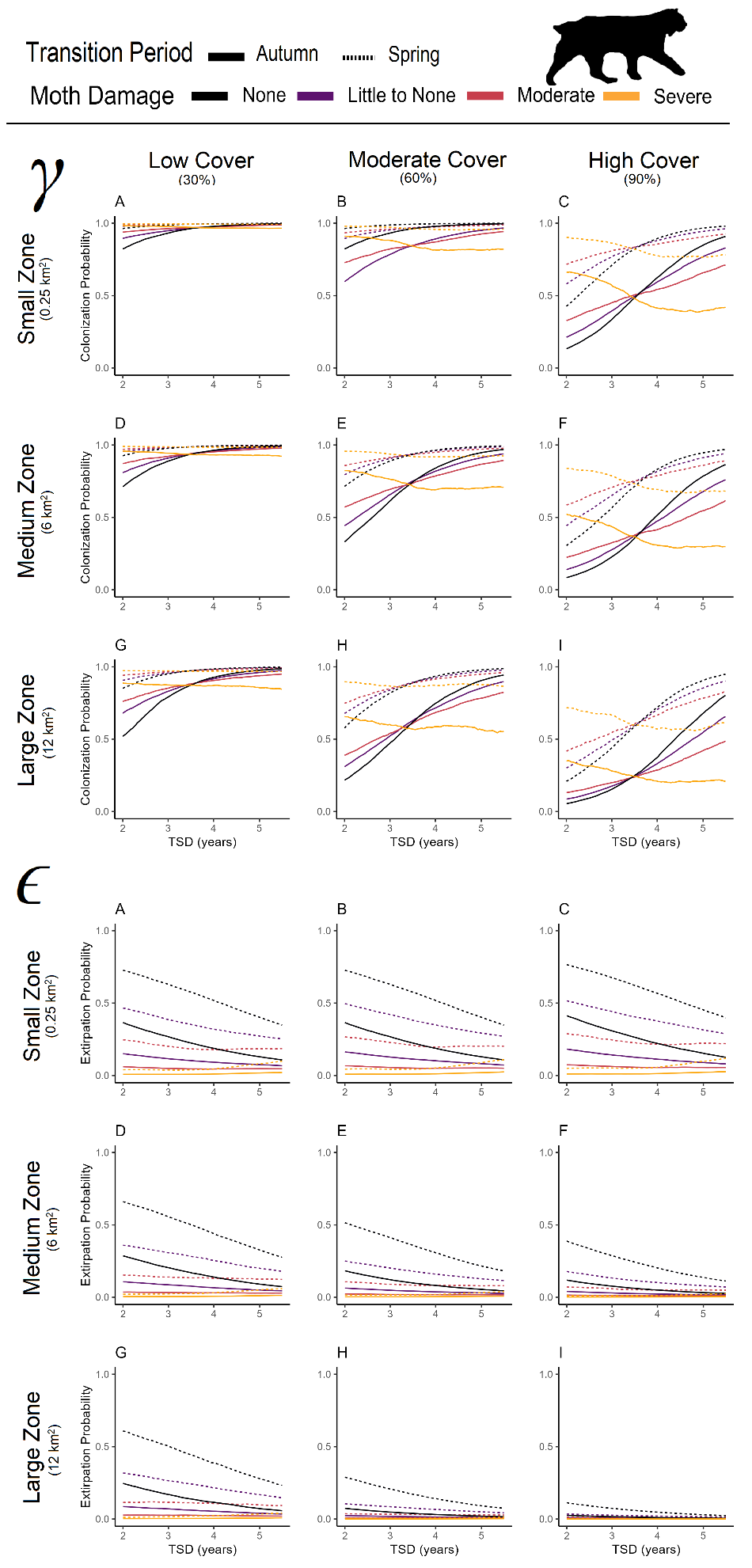


Figure S1. Predicted colonization and extirpation responses of bobcat (*Lynx rufus*) to changes in cover, zone size, transition period, moth damage, and time since disturbance. Each line represents the predicted posterior median.


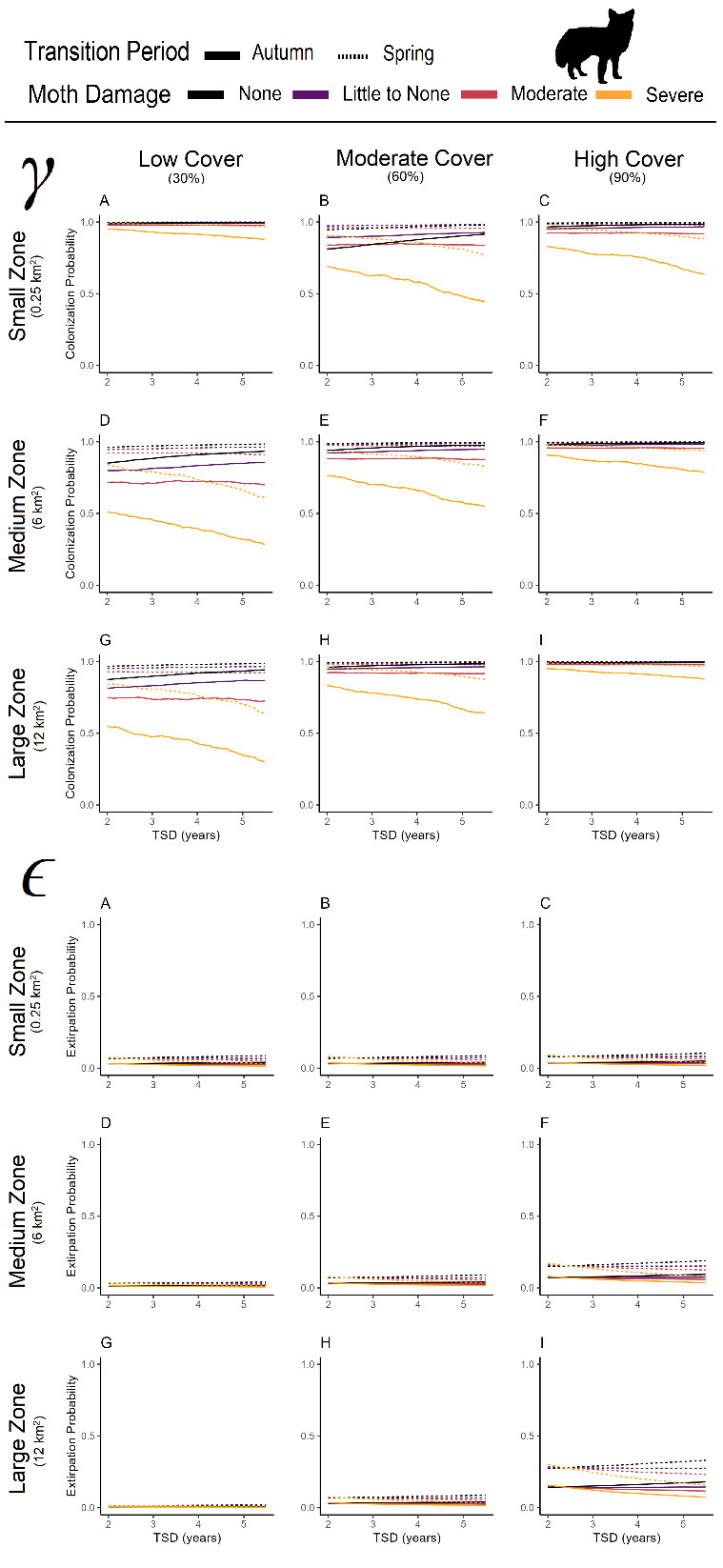


Figure S2. Predicted colonization and extirpation responses of coyote (*Canis latrans*) to changes in cover, zone size, transition period, moth damage, and time since disturbance. Each line represents the predicted posterior median.


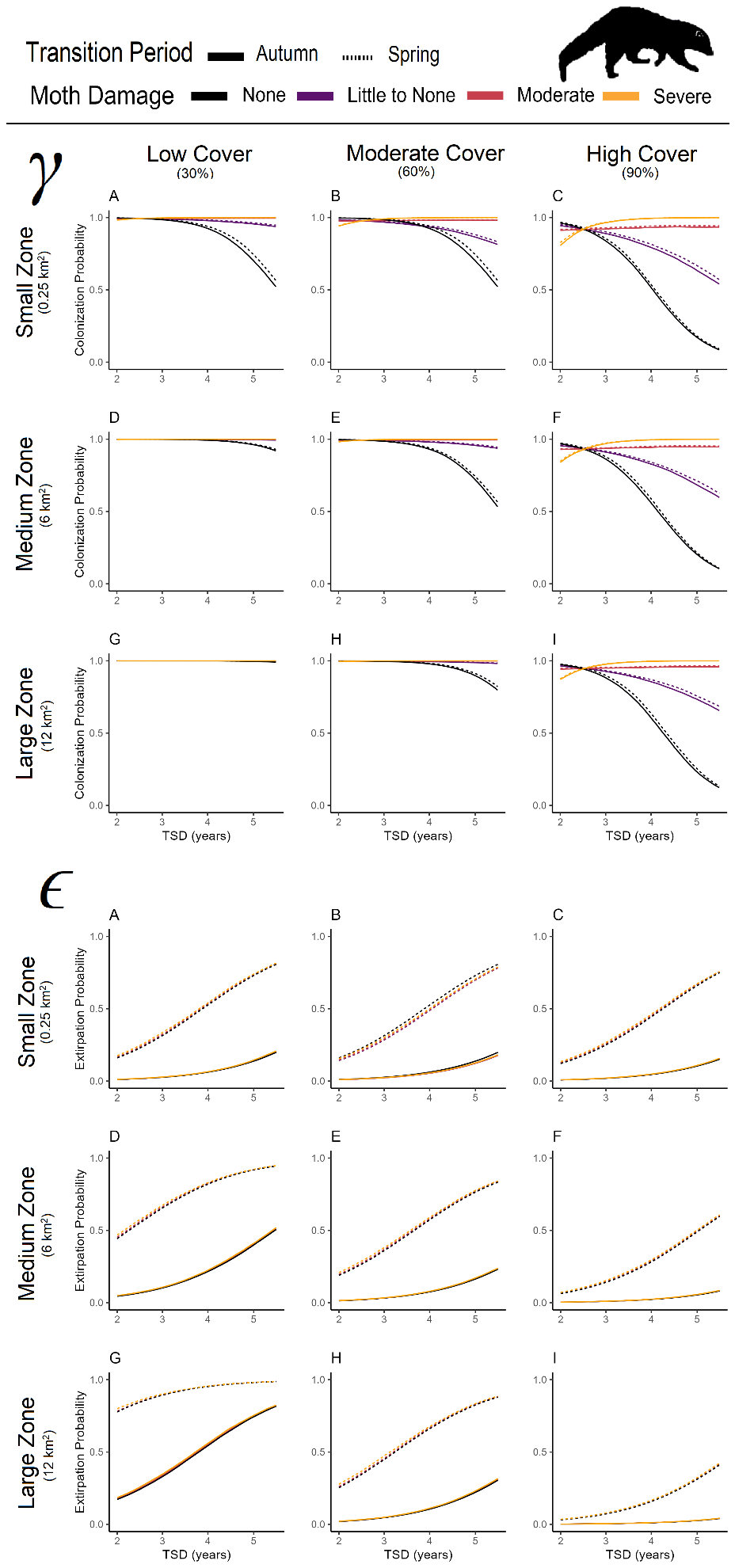


Figure S3. Predicted colonization and extirpation responses of fisher (*Pekania pennanti*) to changes in cover, zone size, transition period, moth damage, and time since disturbance. Each line represents the predicted posterior median.


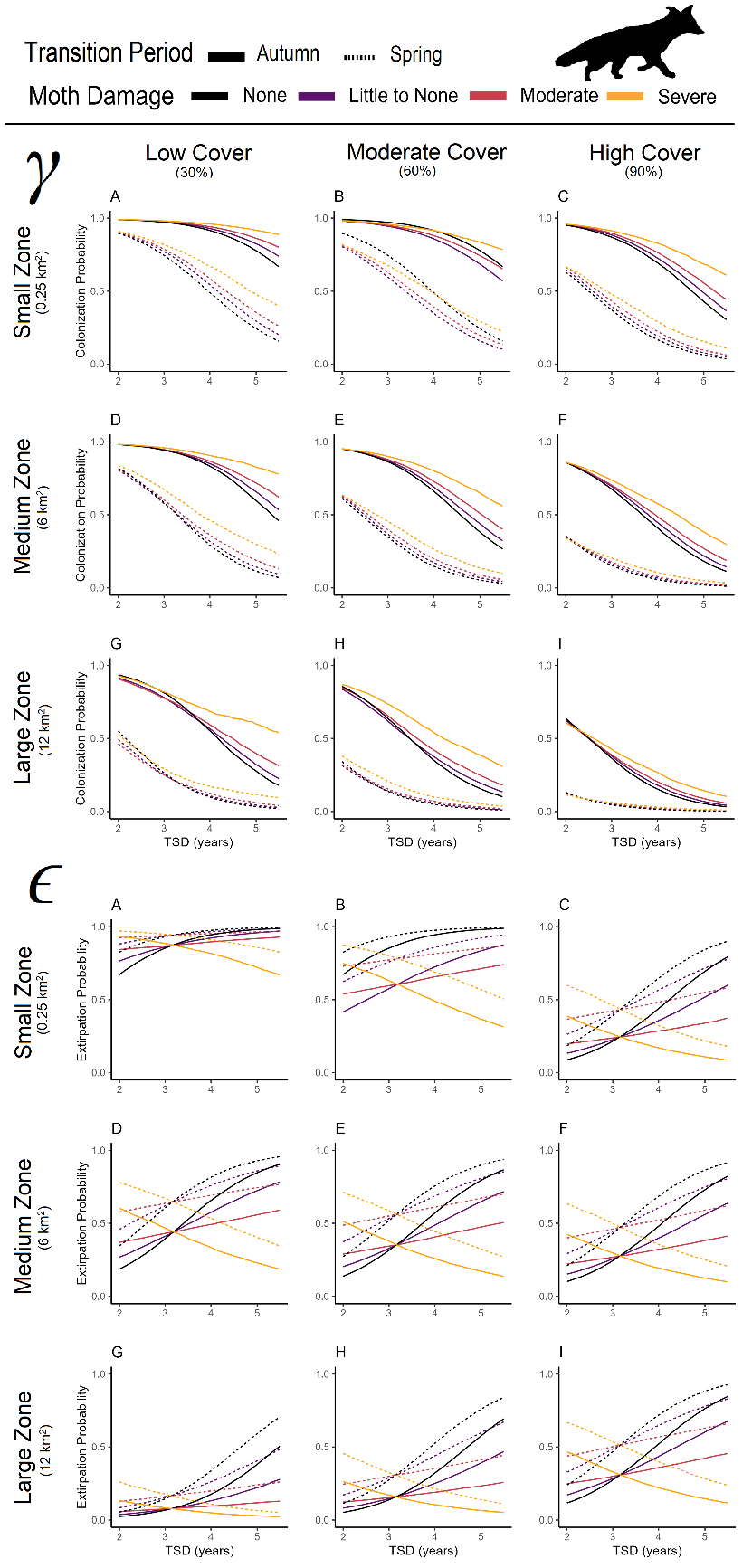


Figure S4. Predicted colonization and extirpation responses of gray fox (*Urocyon cinereoargenteus*) to changes in cover, zone size, transition period, moth damage, and time since disturbance. Each line represents the predicted posterior median.


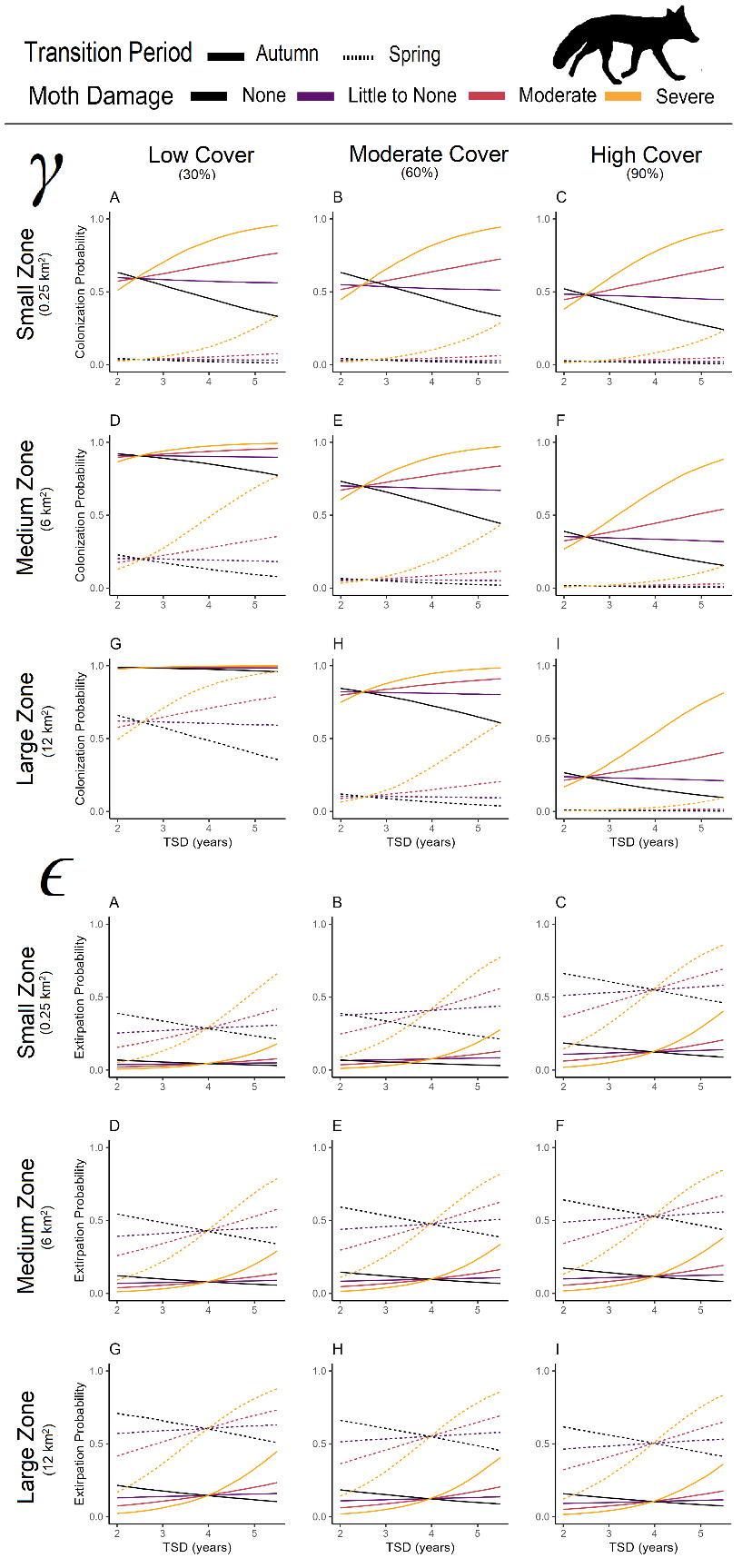


Figure S5. Predicted colonization and extirpation responses of red fox (*Vulpes vulpes*) to changes in cover, zone size, transition period, moth damage, and time since disturbance. Each line represents the predicted posterior median.


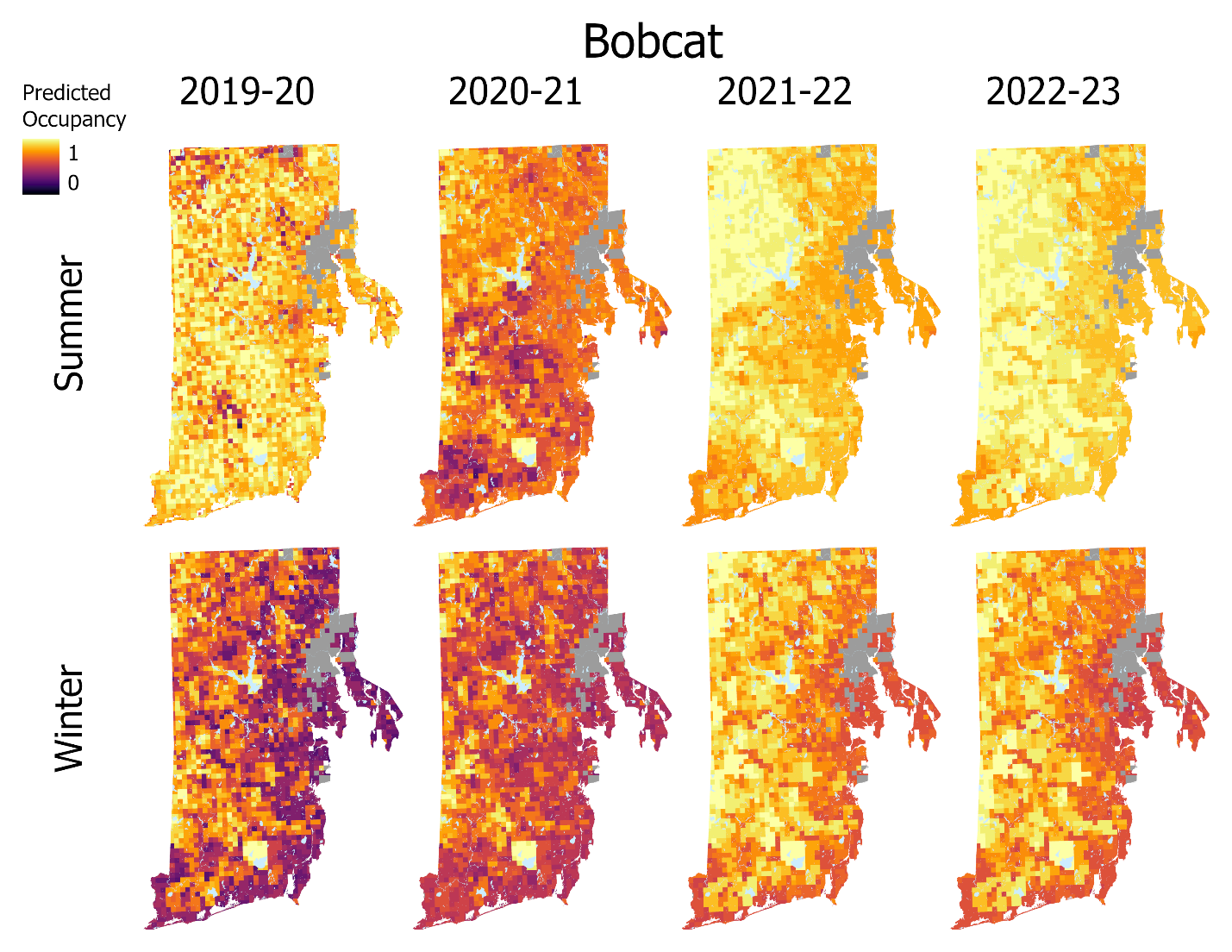


Figure S6. Predicted occupancy across Rhode Island for bobcat from summer of 2019 to winter of 2023.


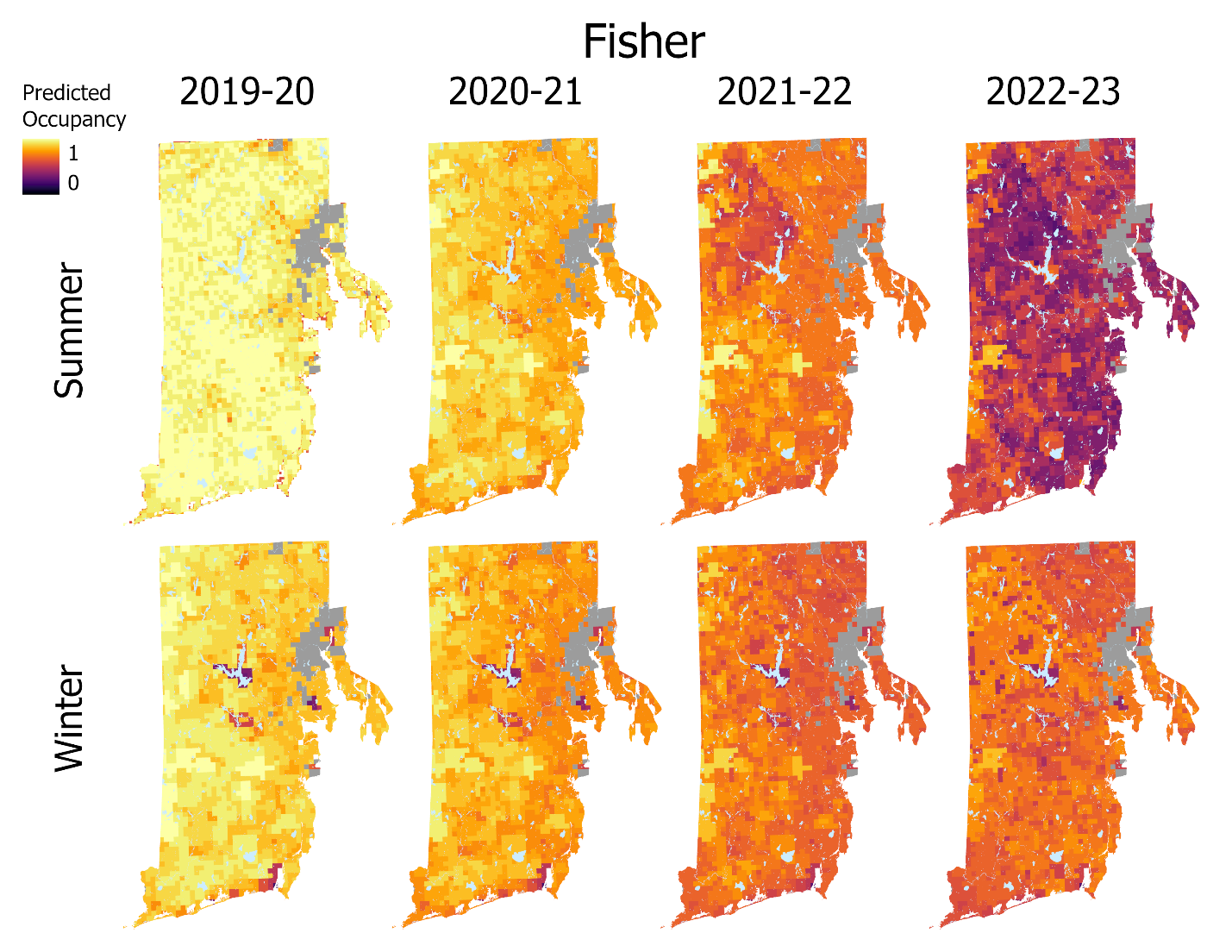


Figure S7. Predicted occupancy across Rhode Island for fisher from summer of 2019 to winter of 2023.


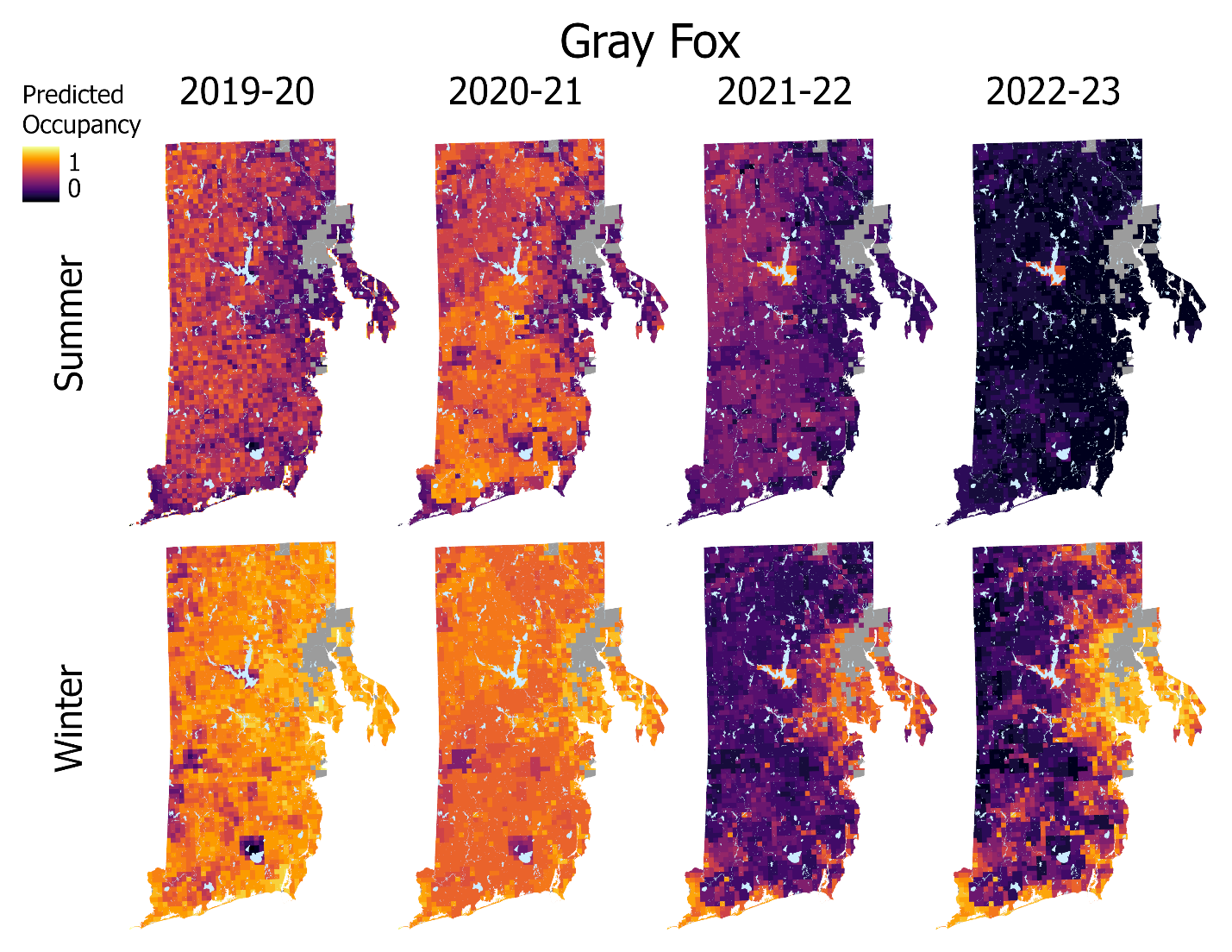


Figure S8. Predicted occupancy across Rhode Island for gray fox from summer of 2019 to winter of 2023.


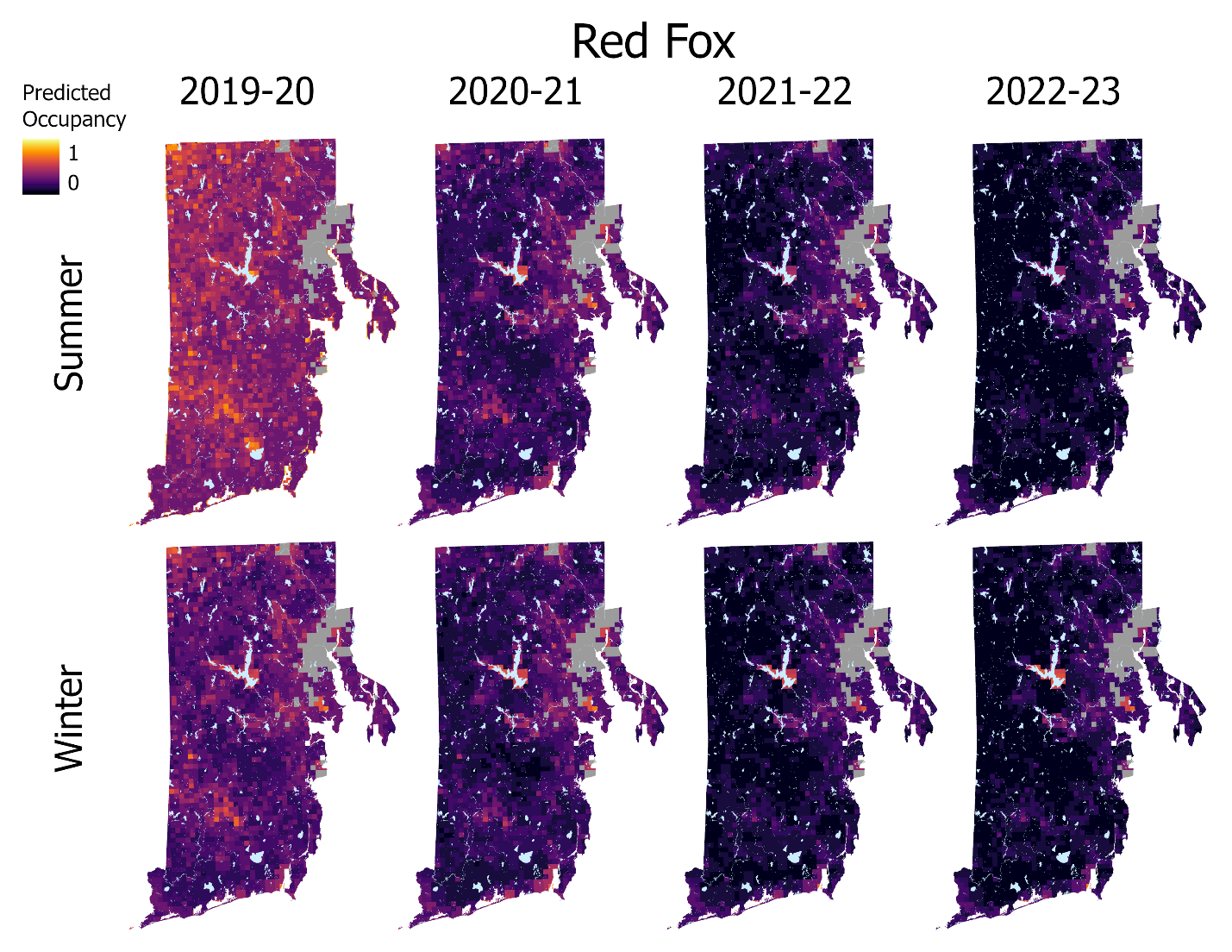


Figure S9. Predicted occupancy across Rhode Island for red fox from summer of 2019 to winter of 2023.
